# Use of machine learning to detect *Escherichia coli* in drinking water in Bangladesh

**DOI:** 10.1371/journal.pone.0343606

**Published:** 2026-08-20

**Authors:** Iqramul Haq, Md. Yusuf Hossain Ador, Diego Nobrega

**Affiliations:** 1 Faculty of Veterinary Medicine, University of Calgary, Calgary, AB, Canada; 2 Department of Agricultural Statistics, Sher-e-Bangla Agricultural University, Dhaka, Bangladesh; 3 Department of Statistics, Jagannath University, Dhaka, Bangladesh; Maulana Abul Kalam Azad University of Technology West Bengal, INDIA

## Abstract

*Escherichia coli (E. coli)* is a key indicator of fecal contamination in freshwater and can signal the presence of other harmful bacteria and viruses. The aim of the study is to evaluate the performance of machine learning (ML) tools to detect *E. coli* in drinking water in Bangladesh using surveillance data under two scenarios: an imbalanced dataset and a balanced dataset. We utilized data from the 2019 Bangladesh Multiple Indicator Cluster Survey, which included a total of 6,069 household drinking water samples. We used agglomerative hierarchical clustering with Ward’s linkage to identify district-level hotspots. Extreme Gradient Boosting with SHapley Additive exPlanations values were used for feature selection, and the Synthetic Minority Over-sampling Technique (SMOTE) was used to address class imbalance in the classification task. We applied nine classical ML models in this study: Adaptive Boosting (AdaBoost), Decision Trees (DT), Gradient Boosting Algorithm (GBA), k-Nearest Neighbors (KNN), Light Gradient-Boosting Machine (LightGBM), Logistic Regression (LR), Naïve Bayes (NB), Random Forest (RF), and Support Vector Machine (SVM), along with a Deep Learning Multi-Layer Perceptron (DL-MLP) model to predict the risk of *E. coli* contamination (REcC) in water. Model performance was evaluated using accuracy, precision, recall, F1 score, Cohen Kappa, area under the curve (AUC), and a violin plot. *E. coli* contamination in drinking water was detected in 39.2% (95% CI: 37.4–41.2) of households. Bandarban district had the highest REcC. After applying SMOTE and 10-fold cross-validation with hyperparameter tuning, model performance was more consistent across algorithms. In terms of model evaluation, AdaBoost slightly outperformed the others with an accuracy of 81.6%, Cohen kappa statistic of 19.4%, precision of 82.2%, recall of 99%, F1-score of 89.8%, and an AUC of 68.6%. Ensemble model for example AdaBoost and GBA models had ability to accurately classify drinking water samples with respect to the presence of *E. coli* using surveillance data than others selected model in this study.

## Introduction

Enterobacteriaceae, particularly *Escherichia coli (E. coli)*, are the dominant commensal bacteria found in the gastrointestinal tracts of humans and other warm-blooded animals, and are also considered important pathogens [1]. Contamination of household drinking water with *E. coli* is a significant public health concern, particularly amongst children [2]. Neonatal meningitis, urinary tract infections, enteritis, septicaemia, and other clinical illnesses are commonly caused by *E. coli* [1].

Over half of the global population uses groundwater as their primary drinking water source [3]. Conflicting demands have led to over-exploitation of 20% of aquifers, with contamination from chemicals, radionuclides, and microorganisms, as well as improper waste and wastewater management, posing significant risks to groundwater supplies [3]. Acute gastrointestinal illnesses (AGIs) brought on by drinking contaminated water spread irregularly throughout location and time as a result of microbiological groundwater contamination events. Contaminated groundwater is the leading cause of waterborne illness in the US, accounting for 64% of drinking water outbreaks between 1989 and 2002, with up to 150 million North American residents relying on groundwater systems [4].

The burden of waterborne illness is increased by the possibility of exposure to enteric pathogens [5]. Enteric infections are more common among sensitive subpopulations, such as the young, old, pregnant, and malnourished. Both low-and-middle income and high-income countries have high rates of infections caused by enteric pathogens. Groundwater contamination can occur in different ways, such as exposure to septic tanks, sewage, municipal wastewater treatment facilities, wildlife, and agricultural residues [5]. Nearly 60% of private well owners in Ireland do not use an appropriate domestic water treatment system [6].

In many countries, storing drinking water is a common practice due to intermittent or lack of direct access to drinking water within homes. Water storage has been identified as an important risk factor for diarrheal diseases [7]. Unhygienic handling during storage can lead to microbial contamination of drinking water [8]. The type of water source also affects contamination levels [9]. Additionally, water supplies may become contaminated in case of open defecation or subpar toilets are used. Social characteristics such as income will also be associated with contamination levels. Households categorised as middle-class or lower-class in Bangladesh were shown to be at a higher risk of drinking water contamination when they obtained their water from sources with elevated levels of *E. coli* [2]. The possibility of increased contamination levels was greatly decreased by treating drinking water, although pet ownership was strongly linked to recontamination.

From a statistical perspective, different approaches were used to identify the presence of *E. coli* contamination among household drinking water. A study in North America used pooled analysis to investigate groundwater contamination in the US and Canada [5]. Another researcher used association rule learning, regression analysis, and variable discretization techniques to identify the relationship between contamination and other predictors [3].

Machine learning (ML) has emerged as a significant solution for various applications such as bioinformatics [10,11], and activity recognition in recent decades. Deep learning (DL) models are powerful tools for computation on large datasets [12]. Since the early 2000s, DL has greatly improved predictive models by automatically extracting and analyzing valuable insights from raw data [13]. A hybrid model is created for general classification tasks, showing better performance than individual ML or DL models [14].

ML techniques were used to investigate the growth of *E. coli* O157:H7 in spiked raw ground beef and evaluated prediction performance using coefficient of determination (R²) and mean squared error [15]. A second study employed an impedimetric electrochemical aptasensor with gold interdigitated electrodes to measure *E. coli* O157:H7 in surface water for hydroponic lettuce irrigation. A ML framework was used to evaluate the effectiveness of existing methods for contamination detection and model performance was assessed using the root mean squared error [16]. Many ML algorithms were used to predict *E. coli* levels such as stochastic gradient boosting, random forest (RF), support vector machines (SVM), and k-nearest neighbor (KNN) [17]. Predictive models for *E. coli* were developed using regression techniques, artificial neural networks (ANN), and two adaptive neuro-fuzzy inference system (ANFIS) structures and their performance was compared using R² and the root mean squared log error [18]. RF, Categorical Boosting, and Naïve Bayes (NB) were used to classify surgery-ward *E. coli* isolates from susceptibility profiles, and performance was evaluated using accuracy, precision, recall, F1-score, and area under the curve (AUC) [19]. RF, logistic regression (LR), and SVM analyzed flow cytometry 2D histograms to detect *E. coli* associated microbial patterns, evaluated by accuracy, sensitivity, precision, and F1-score [20].

Although previous studies identified risk factors for *E. coli* contamination using traditional statistical methods [2,21], this study advances beyond risk factor identification by developing predictive models that classify contamination status in settings where routine laboratory surveillance is absent or infeasible. ML algorithms offer two critical advantages over conventional statistical approaches: (1) higher predictive accuracy through detection of complex non-linear relationships and high-order interactions among environmental, socioeconomic, and household-level predictors, and (2) the capacity to prioritize high-risk locations for targeted sampling when universal testing is not financially or logistically viable. To achieve this, we test the ability of classical ML [Adaptive Boosting (AdaBoost), Decision Trees (DT), Gradient Boosting Algorithm (GBA), KNN, Light Gradient-Boosting Machine (LightGBM), LR, NB, RF, and SVM] and the DL Multi-layer Perceptron (DL-MLP) algorithms using data from the 2019 Multiple Indicator Cluster Survey (MICS) to estimate the risk of *E. coli* contamination (REcC) in the drinking water under two scenarios: (1) an imbalanced dataset assessed using 10-fold stratified cross-validation (CV) without hyperparameter tuning, and (2) Synthetic Minority Over-sampling Technique (SMOTE)-balanced training data assessed using 10-fold stratified CV with hyperparameter tuning. We also identified the relevant predictors. The novelty of this work lies in transitioning from explanatory risk factor analysis to operational prediction tools that enable resource-constrained health systems to allocate limited diagnostic capacity based on model-predicted contamination probability rather than reactive testing. The Bangladeshi government is implementing advanced analytical approaches to predict *E. coli* contamination risk, aiming to improve water quality and protect public health. The 2019 MICS data can help identify hotspots and vulnerable populations, enhancing policymakers’ understanding of socio-demographic factors influencing contamination risk. This evidence-based approach can inform targeted interventions and resource allocation.

## Methods

### Data source and study design

The study analyzed secondary dataset from the 2019 MICS in Bangladesh to assess *E. coli* concentrations in drinking water. A two-stage, stratified cluster sampling method was implemented. Primary sampling units were selected from enumeration areas (EAs) defined by the 2011 Bangladesh Population and Housing Census in the first stage. Subsequently, 20 households (HHs) were selected from each EA via systematic random sample, with four households chosen for arsenic level assessment in their drinking water. Additionally, two HHs were randomly selected to measure *E. coli* concentrations in both household drinking water and its source. The study targeted a sample size of 6,440 households, with 6,069 (98.7%) successfully providing data for *E. coli* testing [22].

### Outcome variable

In this paper, REcC in the HH drinking water is the response variable. The most recommended indicator for fecal contamination is the number of *E. coli* bacteria counts in a 100 mL sample. The outcome variable is *E. coli* contamination in household drinking water, which is categorized using WHO criteria into low (<1 colony forming units [CFU]/100mL), medium (1–10 CFU/100mL), and high risk (11–100 or more CFU/100 mL) categories [23,24]. To this study, medium and high-risk categories were grouped as increased risk category. Specifically, households with 1 or more CFU per 100 mL of drinking water were categorized as ‘Yes’ for the REcC, while those with less than 1 CFU per 100 mL were categorized as ‘No’ for the REcC. Mathematically, the outcome variable can be defined as:


$$\begin{array}{c} \text{REcC}=\;\left\{ \begin{array}{cc} \text{Yes},\; & \text{If CFU}\geq \text{1}\text{ per 100 mL of drinking water}\\ \text{No},\; & \text{If CFU}<\text{1}\text{ per 100 mL of drinking water} \end{array}\right. \end{array}$$

### Independent variables

In addition to the outcome variable, division, place of residence, age of household head, household head education, household size, wealth index, livestock ownership, types of toilet facility, sources of drinking water, location of the water source, treatment of drinking water, place of handwashing, mass media exposure, and women functional difficulties were selected as potential factors influencing REcC among drinking water in households (Table 1).

**Table 1:** Summary of the selected covariates.

Variables	Categorization
Division	Barishal, Chattogram, Dhaka, Khulna, Mymensingh, Rangpur, Rajshahi, Sylhet
Place of residence	Urban or Rural
Age of household head	15-64, 65+
Household head education	No education, Primary, Secondary+
Household size	<4, 4-5, 6+
Wealth index	Poor, Middle, Rich
Livestock ownership	Yes or No
Types of toilet facility	Flush, Pit latrine, Others
Sources of drinking water	Piped water, Tube wall, Others
Location of the water source	Own dwelling, Own yard/plot, Elsewhere
Treatment of drinking water	Yes or No
Place of handwashing	Dwelling, Yard/plot, Mobile object, No place
Mass media exposure	Yes or No
Women functional difficulty	Yes or No

### Statistical analysis

We first used a traditional approach based on Chi-squares tests to explore associations between REcC and explanatory variables. Agglomerative hierarchical clustering using Ward’s linkage was used to identify district-level hotspots for the REcC in drinking water. This method automatically groups data points into clusters based on their similarities, starting with each data point in its own cluster and merging the closest two until a single cluster remains.

### XGBoost SHapley additive explanations (SHAP)

Prior to the ML analysis, a predictive model for imbalanced classification was developed using eXtreme Gradient Boosting (XGBoost), with the goal of reducing overfitting and improving accuracy through high speed [25,26]. We then used SHapley Additive exPlanations (SHAP) to interpret XGBoost model predictions, providing a comprehensive understanding of each feature’s contribution to the model’s predictions [27]. The SHAP summary plot was used to rank predictors by their mean absolute SHAP values (average absolute impact on the XGBoost output). We then dropped predictors with near-zero SHAP contribution and refit the model using the remaining variables to reduce noise and overfitting. Next, we reviewed SHAP dependence plots to identify clear non-linear patterns and strong interactions among key predictors. Based on these patterns, we updated feature coding where needed (for example, collapsing sparse categories and using more appropriate transformations) and refit the final model. Model performance was re-assessed using the same validation procedure to confirm that these SHAP-guided changes improved generalization and produced more stable predictions.

Additionally, Cramer’s V correlation was used to identify potential collinearity among features, ranging from 0 (no association) to 1 (perfect association) [28]. Values between 0.36 and 0.49 indicated a substantial correlation, while values of 0.50 or higher were considered strong correlation [29].

### ML classifiers

This study employed a comprehensive approach, integrating nine (09) classical ML models and one (01) DL techniques, to predict the REcC in household drinking water in Bangladesh. In the training dataset, we applied nine classical ML models utilized in this study include AdaBoost, DT, GBA, KNN, LightGBM, LR, NB, RF, and SVM. Additionally, we incorporated DL-MLP model into our analysis. ML models and evaluation metrics are extensively utilized in the literature for classification tasks [25,30–37].

### Data preparation and model evaluation

**Fig 1:**
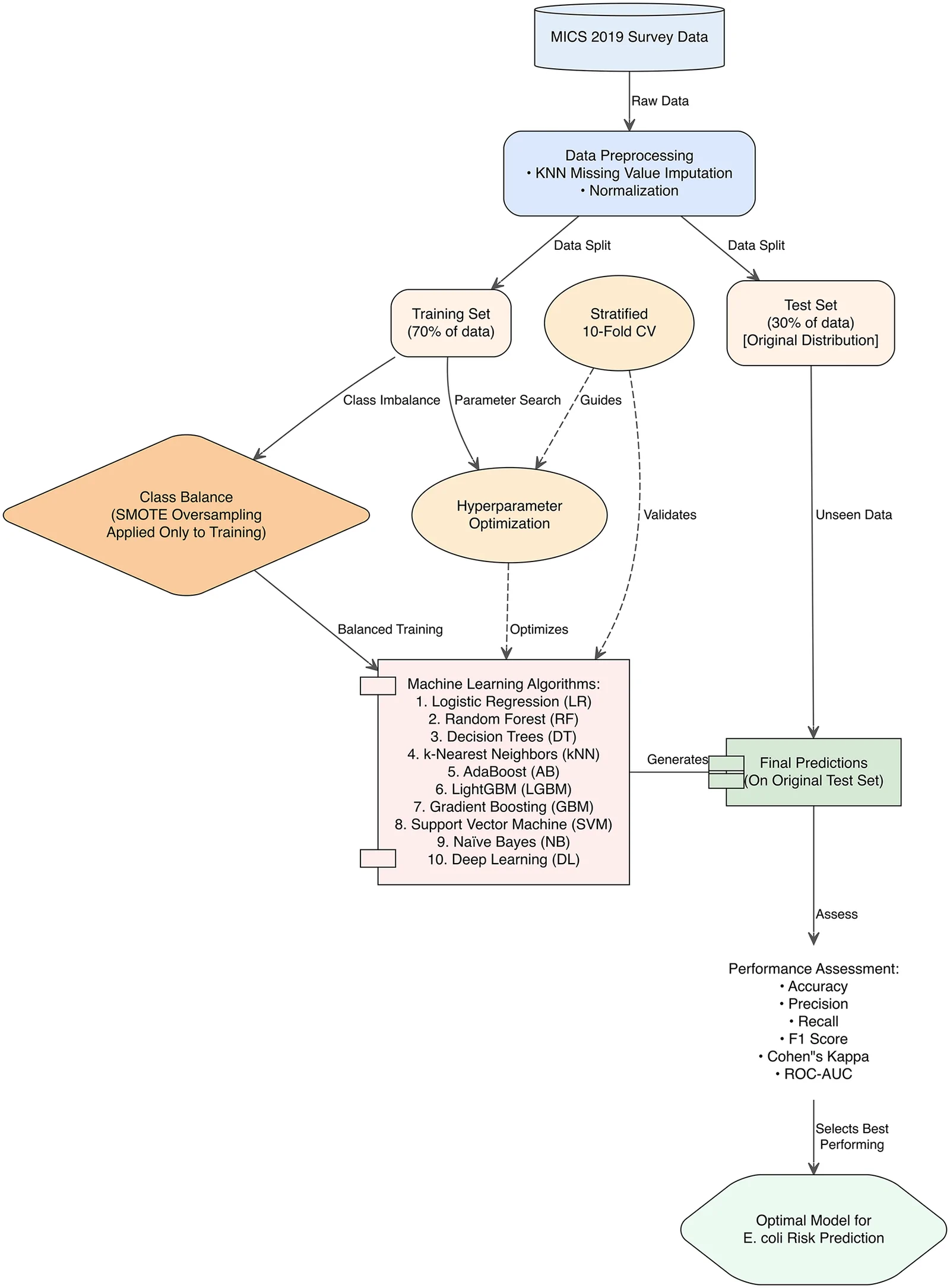
Flow chart of the procedure of the nine ML classifiers and one DL classifier.

For the ML approach, the MICS dataset was split into training (70%) and test (30%) sets using stratification to preserve the REcC class distribution. The dataset was checked for missing values, which were handled using KNN imputation [38] before training the selected ML and DL algorithms was implemented within a scikit-learn *Pipeline* so that the same steps were applied consistently during training and validation. Data preprocessing was performed using *Python* [35], *Scikit-learn* [39], *pandas* [40] and *Keras* [41]. Imbalanced data refers to unequal representation of classes in classification problems, including binary and multi-class tasks [42]. The training set was subjected to 10-fold CV to identify optimal hyperparameters. We used the test dataset to evaluate the performance of models measured by various evaluation parameters, including accuracy, precision, recall, F1 score, and AUC [33,34,43–46]. Results were presented in both tabulated and graphical formats, with violin plots [33] being used to illustrate the distribution of model performances. The overall procedure of the ML approach is displayed in Fig 1.

### Classification outputs

After training, each household sample was classified using two model outputs. Predicted class labels were obtained using predict(X) and used to generate the confusion matrix and compute accuracy, precision, recall, F1-score, and Cohen’s kappa. When available, predicted probabilities were obtained using predict_proba(X)[:,1]; for SVM, probability estimation was enabled (probability = True) to obtain probability outputs. Receiver operating characteristic (ROC)-AUC was computed using predicted probabilities, whereas the other metrics were computed using predicted class labels.

### Hyperparameter

Hyperparameters control the learning process and help determine the best model parameters [47]. A ML model’s performance depends on optimal hyperparameters, which can be tuned using methods like *grid search*, *random search*, and *Bayesian optimization* [48]. Grid search divides the hyperparameter space into a grid and evaluates all combinations using CV to find the optimal values [49]. We used grid search with CV to optimize each model’s hyperparameters. We optimized model hyperparameters using *GridSearchCV* applied to the training set only. For each algorithm, we evaluated the parameter grids listed in S1 Table in S1 File. Model selection was performed using stratified 10-fold CV (*StratifiedKFold*, *n* = 10, shuffle = True, random_state = 42) and the mean F1-score was used as the tuning criterion. The best model (*best_estimator_*) was refitted on the full training set and evaluated once on the held-out test set.

### SMOTE

Class imbalance arises when one class has far more samples than another, causing models to overfit the majority class and perform poorly on the minority class [50–52]. SMOTE generates synthetic minority-class samples to balance the dataset, reducing majority-class bias and helping the classifier learn the minority class more effectively [53–55]. Since the outcome variable (REcC in drinking water) was imbalanced (39.2% “Yes” and 60.8% “No”), we used the SMOTE to address class imbalance in this binary outcome [56]. After SMOTE, synthetic samples are generated for the minority class so that the class distribution becomes more balanced, with roughly equal numbers of instances in each class [57]. Several studies have shown that SMOTE usually boosts minority-class precision, recall, and F1-score, giving a more balanced and fair assessment of model performance [52,57]. For our analysis, we evaluated two training scenarios for each model: (i) the original imbalanced training data, assessed using 10-fold stratified CV without hyperparameter tuning, and (ii) SMOTE-balanced training data, assessed using 10-fold stratified CV with hyperparameter tuning. When SMOTE was used, it was applied only to the training split within each CV fold; the validation folds and the final held-out test set remained non-synthetic.

## Results

### Prevalence of *E. coli* in household drinking water

The overall prevalence of *E. coli* contamination in drinking water was 39.2% (95% CI: 37.4 to 41.2).

### Association between sociodemographic factors and *E. coli* contamination

In univariate analysis, the REcC in drinking water in Bangladesh was significantly associated with several factors, including division, residence, household head education, household size, wealth index, livestock ownership, types of toilet facilities, sources of drinking water, treatment methods for drinking water, handwashing locations, mass media exposure, and women functional difficulties (p < 0.05) (Table 2).

**Table 2:** Characteristics of enrolled households according to REcC in drinking water.

Variables	REcC		$\chi^{2}\;$(*p*-value)
	Yes (%)	No (%)	
**Division**			
Barishal	15.8	84.2	353.92 (<0.001)
Chattogram	49.7	50.3	
Dhaka	51.4	48.6	
Khulna	37.1	62.9	
Mymensingh	43.4	56.6	
Rangpur	23.4	76.6	
Rajshahi	27.5	72.5	
Sylhet	37.1	62.9	
**Place of residence**			
Urban	46.9	53.1	43.10 (<0.001)
Rural	37.0	63.0	
**Age of household head (in years)**			
15-64	39.0	61.0	0.577 (0.447)
65+	40.4	59.6	
**Household head education**			
No education	42.3	57.7	13.89 (0.001)
Primary	37.9	62.1	
Secondary+	37.0	63.0	
**Household size**			
<4	36.2	63.8	13.78 (0.001)
4-5	40.2	59.8	
6+	42.2	57.8	
**Wealth index**			
Poor	37.6	62.4	19.69 (<0.001)
Middle	35.9	64.1	
Rich	42.6	57.4	
**Livestock ownership**			
Yes	39.5	60.5	24.81 (<0.001)
No	42.8	57.2	
**Types of toilet facility**			
Flush	40.0	60.0	3.554 (0.045)
Pit latrine	38.6	61.4	
Others	36.1	63.9	
**Sources of drinking water**			
Piped water	54.4	45.6	186.70 (<0.001)
Tube wall	36.0	64.0	
Others	77.6	22.4	
**Location of the water source**			
Own dwelling	37.9	62.1	0.242 (0.886)
Own yard/plot	37.3	62.7	
Elsewhere	38.0	62.0	
**Treatment of drinking water**			
Yes	58.0	42.0	102.58 (<0.001)
No	37.1	62.9	
**Place of handwashing**			
Dwelling	45.1	54.9	19.56 (<0.001)
Yard/plot	38.0	62.0	
Mobile object	39.7	60.3	
No place	36.6	63.4	
**Mass media exposure**			
Yes	40.7	59.3	7.41 (0.007)
No	37.3	62.7	
**Women functional difficulty**			
Yes	31.5	68.5	4.889 (0.027)
No	40.1	59.9	

The highest percentage of positive households was observed in Dhaka division (51.4%), followed by Chattogram division (49.7%), Mymensingh division (43.4%), Khulna and Sylhet divisions (both at 37.1%), Rajshahi division (27.5%), Rangpur division (23.4%), and Barishal division, which had the lowest risk (15.8%). The proportion of positive households was higher in urban areas (46.9%) than rural areas (37%). There was a significant association between education level of household head and REcC, with 42.3% of households in Bangladesh having heads with no formal education. Approximately 42.2% of households with more than six members were positive for *E. coli* contamination in their drinking water. With respect to social-economic characteristics, most houses positive for *E. coli* contamination (42.6%) were of high-income families. Livestock ownership and toilet facilities were significant factors for *E. coli* contamination risk among households in Bangladesh. Households with flush toilet facilities had increased prevalence of *E. coli* contamination compared to those with other facilities. A larger proportion (58%) of households that treated their drinking water were at increased REcC. Additionally, a higher percentage of households where hands were washed inside the dwelling (45.1%) were at REcC, followed by those washing hands in the yard/plot (38%), on a mobile object (39.7%), and those with no designated place for hand washing (36.6%). Households with exposure to mass media had a higher REcC (40.7%) compared to those without exposure to mass media (37.3%) (Table 2).

### District-wise REcC

**Fig 2:**
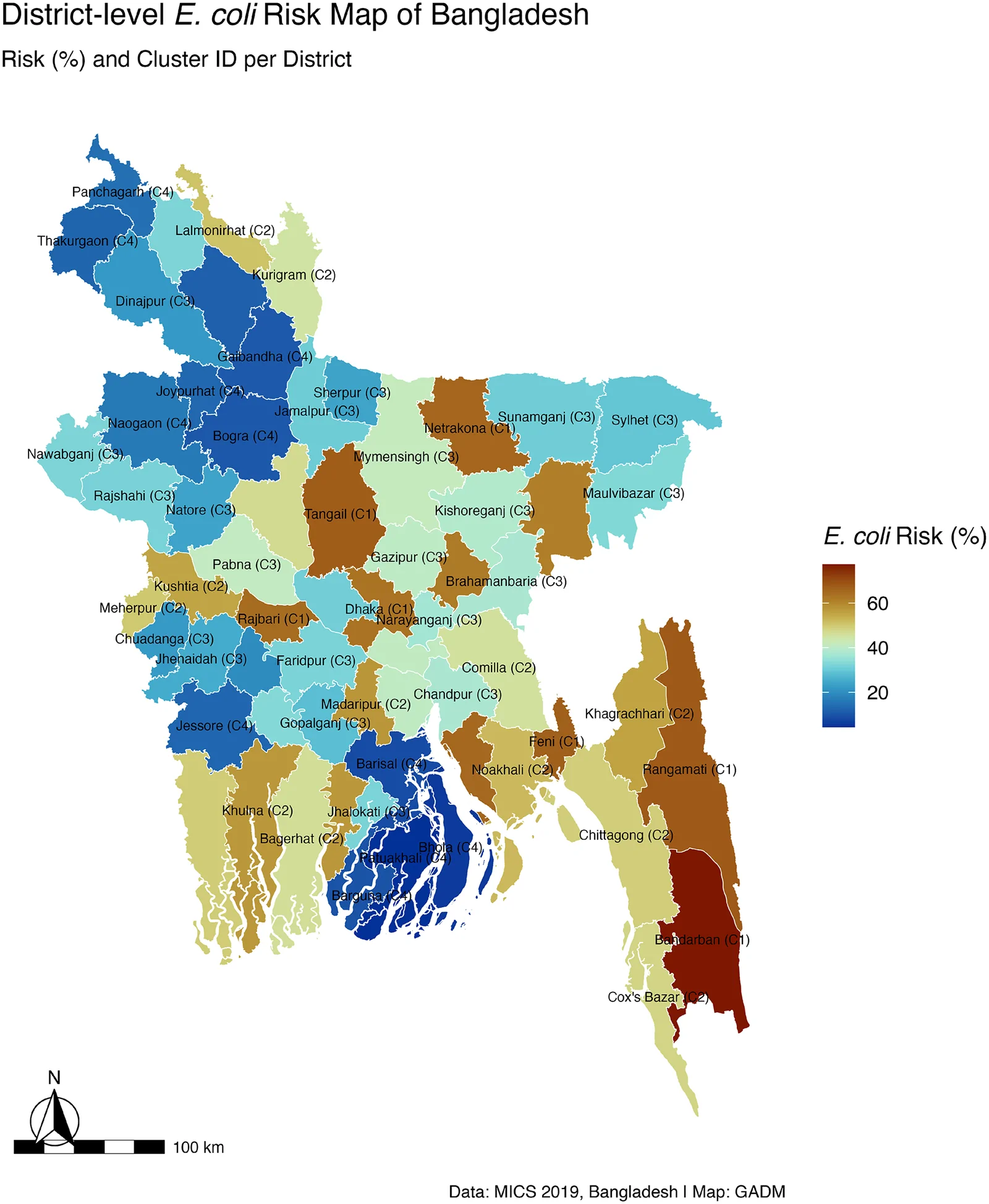
District-wise map of the proportion of *E. coli* contamination in household drinking water across Bangladesh, generated using agglomerative hierarchical clustering with Ward linkage. Administrative district boundaries were obtained from the GADM database (Bangladesh ADM2) and the map was generated by the authors in R using sf and ggplot2 without any proprietary basemap tiles. Data: MICS 2019, Bangladesh. Map: GADM (ADM2).

Certain districts in Bangladesh had higher REcC compared to others. We detected geographical clustering in contamination results using agglomerative hierarchical clustering, as shown in the dendrogram in S1 Fig in S1 File. The clustering was based on data from all sixty-four districts affected by *E. coli* in drinking water. The analysis resulted in four distinct clusters, with sizes of 10, 15, 12, and 27 districts, respectively, which were sorted according to the level of contamination of drinking water. Cluster C1 contained districts with relatively high REcC (up to 60%), whereas districts in cluster C4 had low REcC (around 20%). Cluster 1 (highest prevalence of contamination) comprises Bandarban, Dhaka, Habigonj, Narsingdi, Feni, Tangail, Rangamati, Rajbari, Lakshmipur, and Netrokona. Cluster 2 includes Khagrachari, Kushtia, Khulna, Madaripur, Pirojpur, Noakhali, Chattogram, Satkhira, Lalmonirhat, Meherpur, Cumilla, Kurigram, Cox’s Bazar, Bagerhat, and Sirajganj. Cluster 3 covers Jessore, Joypurhat, Rangpur, Thakurgaon, Naogaon, Panchagarh, Bhola, Patuakhali, Barisal, Barguna, Bogura, and Gaibandha. Cluster 4 consists of all remaining districts not categorized in the first three clusters (Fig 2).

Fig 2 shows the district level variation of REcC in the drinking water using agglomerative hierarchical cluster approach. High REcC was observed in Bandarban, Dhaka, Habigonj, Narshingdi, Feni, Rangamati, Tangail, Rajbari, Lakshmipur, and Netrokona districts, which incorporated the first cluster with > 58% of households being positive for *E. coli*.

### Feature selection

**Fig 3:**
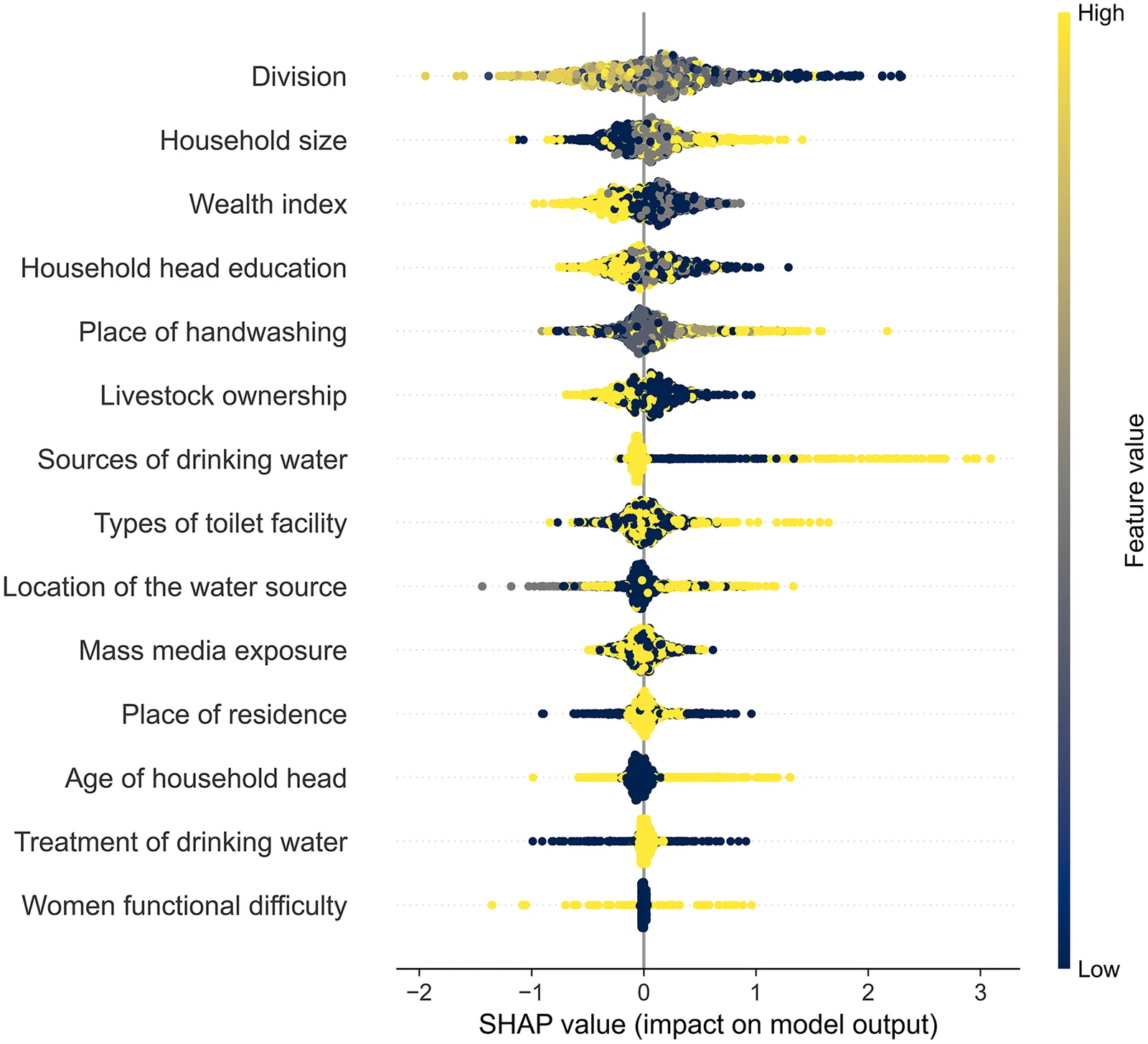
Feature importance based on SHAP values. The beeswarm summary plot displays the distribution of SHAP values for the top predictors of REcC in household drinking water, with features ordered by their overall contribution to the model.

Fig 3 displays SHAP values and top fourteen features with highest impact, with yellow indicating high and dark blue indicating low, with horizontal axis representing each feature’s contribution to prediction. Positive SHAP values indicate higher contamination risk, while negative values indicate lower risk; for example, higher division differences were associated with increased contamination. Regarding SHAP, geographical division was found to be the most significant predictor, followed by the household size, wealth index, household head’s education level, place of handwashing, and livestock ownership.

### Cramér’s V correlation

Cramér’s V correlation analysis revealed strong associations between various factors, particularly the place of handwashing and the location of the water source. Consequently, we excluded the place of handwashing from further ML modeling to predict *E. coli* levels in water (S2 Fig in S1 File).

The distribution of drinking-water sources across divisions is presented in S3 Fig in S1 File. There was a significant association between division and drinking-water source (χ² = 497.52, p < 0.001). Tubewell water was the predominant source in every division, whereas the use of piped water varied by region. Piped water use was highest in Dhaka (20.5%) and substantially lower in the other divisions (2.5%−9.1%). This geographic variation in water-source profiles is consistent with the SHAP results, which identified both division and drinking-water source as important predictors of *E. coli* contamination risk.

### REcC in the drinking water using ML models

**Fig 4:**
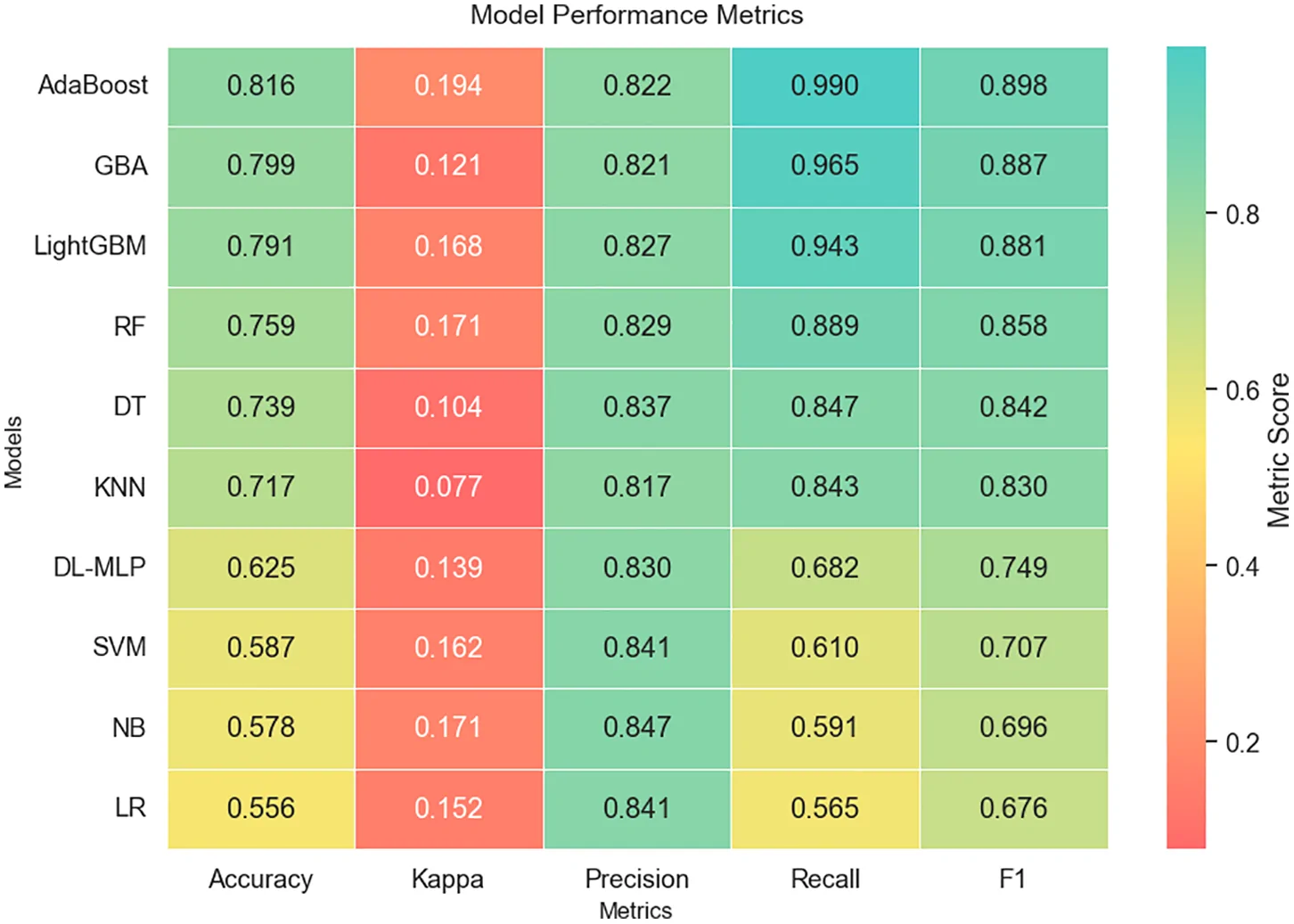
Classification performance measure of the algorithms and comparison. Performance indicators (accuracy, Cohen’s kappa coefficient, precision, recall, and F1 score) for ML algorithms (SVM, RF, NB, LR, LightGBM, KNN, GBA, DT, AdaBoost and DL-MLP).

For the ML models, we tested nine algorithms RF, LR, DT, GBA, AdaBoost, LightGBM, KNN, NB, and SVM and as well as a DL approach based on an DL-MLP. Model performance after applying SMOTE and CV with hyperparameter tuning is presented in Fig 4. The corresponding results for imbalanced dataset including 10-fold CV (before SMOTE and before hyperparameter tuning) are provided in the Supplementary Materials (S4 and S5 Figs in S1 File). The predictive performance of each algorithm was compared based on accuracy, Cohen’s Kappa (κ), precision, recall, and F1-score. In both scenarios, scenario 2 (SMOTE-balanced training with CV and hyperparameter tuning) performed better than scenario 1 (original imbalanced training data).

For scenario 2 (after SMOTE), AdaBoost demonstrated the highest accuracy at 81.6%, correctly predicting the REcC in household drinking water (Fig 4). It also achieved the highest Cohen’s Kappa (κ = 0.194), recall (0.990), and F1-score (0.898). GBA and LightGBM performed similarly well, with accuracies of 0.799 and 0.791, respectively, and strong balance between sensitivity and precision (GBA: recall 0.965, F1 0.887; LightGBM: recall 0.943, F1 0.881). RF and DT showed moderate-to-strong performance (RF: accuracy 0.759, F1 0.858; DT: accuracy 0.739, F1 0.842), while KNN achieved reasonable accuracy (0.717) and F1 (0.830) but had the lowest κ (0.077), indicating weaker improvement over chance agreement.

In contrast, LR and NB had lower accuracies (0.556 and 0.578) and lower F1-scores (0.676 and 0.696), despite relatively high precision (both ≥ 0.841). SVM also showed low accuracy (0.587) and a comparatively low F1-score (0.707). The DL-MLP model showed intermediate performance (accuracy 0.625, recall 0.682, F1 0.749). Among the ten classifiers, the AdaBoost ML classifier slightly outperformed the others (Fig 4 and S6 Fig in S1 File).

**Fig 5:**
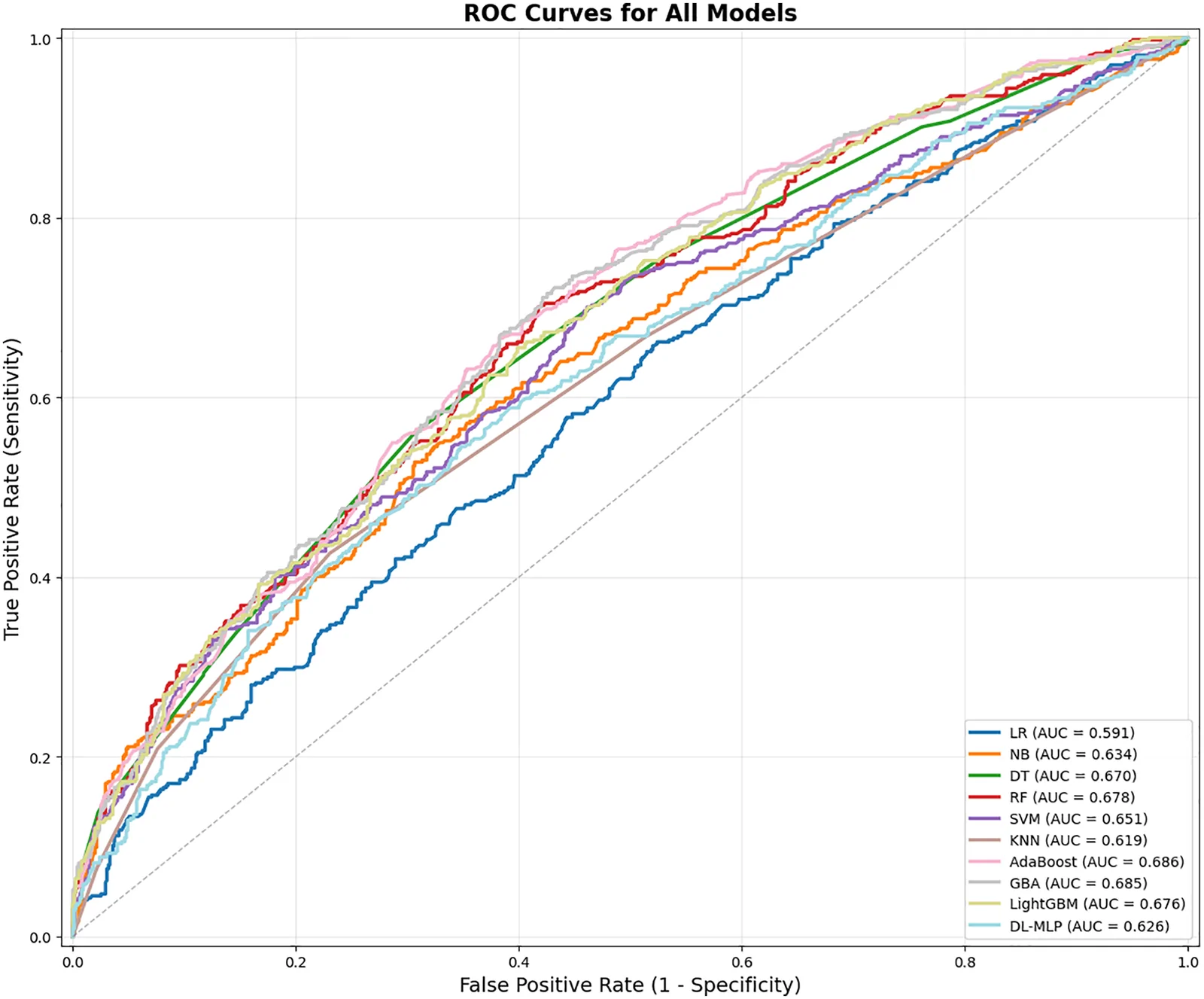
ROC curves and AUC for ten classifiers (AdaBoost, DL-MLP, DT, GBA, KNN, LightGBM, LR, NB, RF, and SVM) using 10-fold CV to predict the REcC in household drinking water.

To predict the REcC among households in Bangladesh, the AUC measure was estimated for the LR, RF, GBA, DT, DL-MLP, KNN, AdaBoost, LightGBM, SVM and NB model models (Fig 5). The estimated AUC values were 0.591 (LR), 0.634 (NB), 0.670 (DT), 0.678 (RF), 0.651 (SVM), 0.619 (KNN), 0.686 (AdaBoost), 0.685 (GBA), 0.676 (LightGBM), and 0.626 (DL-MLP). Overall, the boosting and tree-based models showed the best discrimination for predicting REcC in household drinking water. AdaBoost achieved the highest AUC (0.686), followed closely by GBA (0.685), RF (0.678), and LightGBM (0.676). In contrast, LR had the lowest AUC (0.591), indicating the weakest discriminative performance among the evaluated models.

**Fig 6:**
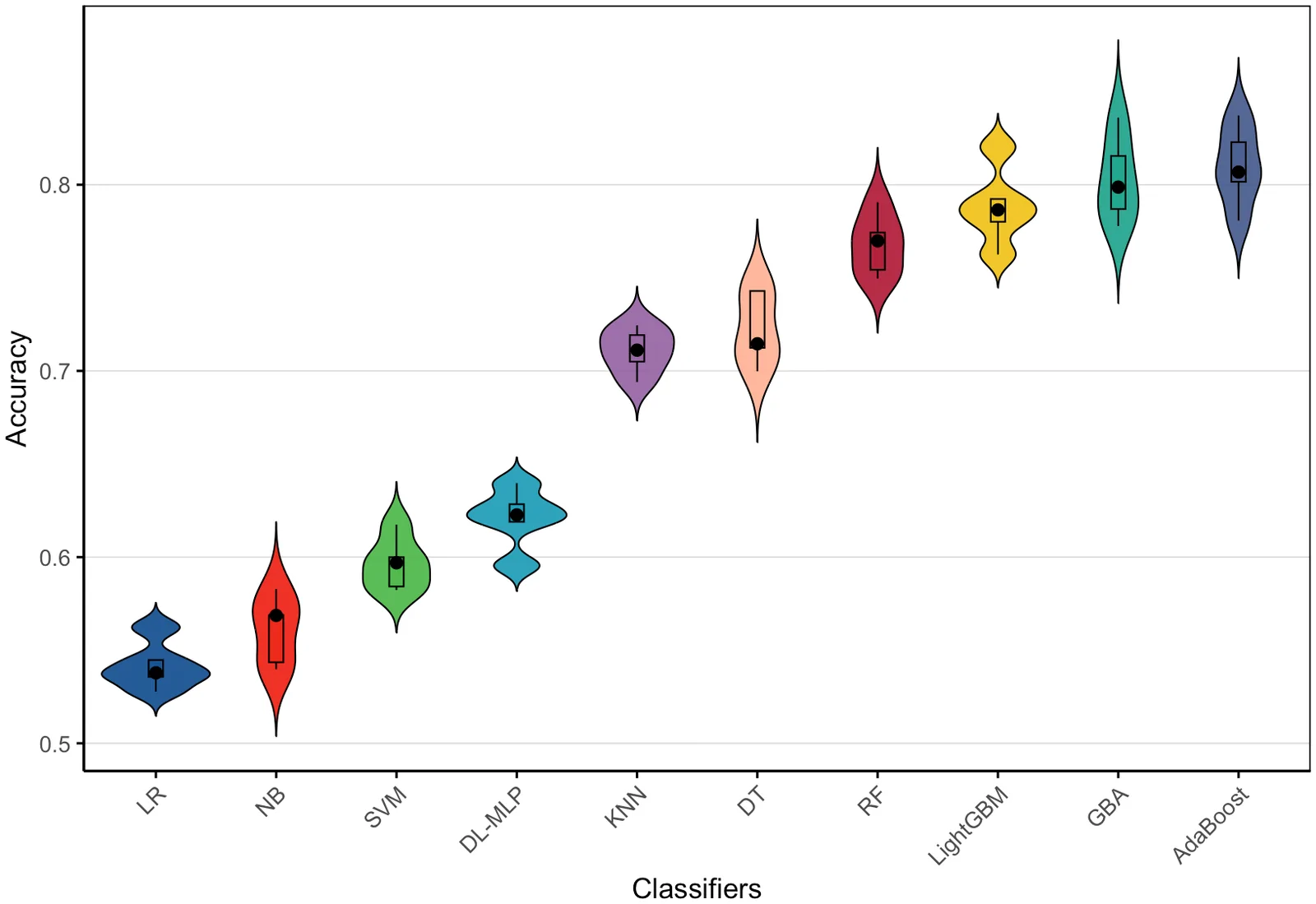
Violin plots showing the distribution of accuracy across 10-fold CV after SMOTE and hyperparameter tuning for ten classifiers (AdaBoost, DL-MLP, DT, GBA, KNN, LightGBM, LR, NB, RF, and SVM) used to predict the REcC in household drinking water. The central point indicates the median and the inner band shows the interquartile range.

AdaBoost model achieved the highest and most consistent median accuracy across folds, followed by GBA, LightGBM, and RF. Unlike a box plot, the violin plot in Fig 6 displays the full distribution of the 10-fold CV accuracy results.

### Feature importance

**Fig 7:**
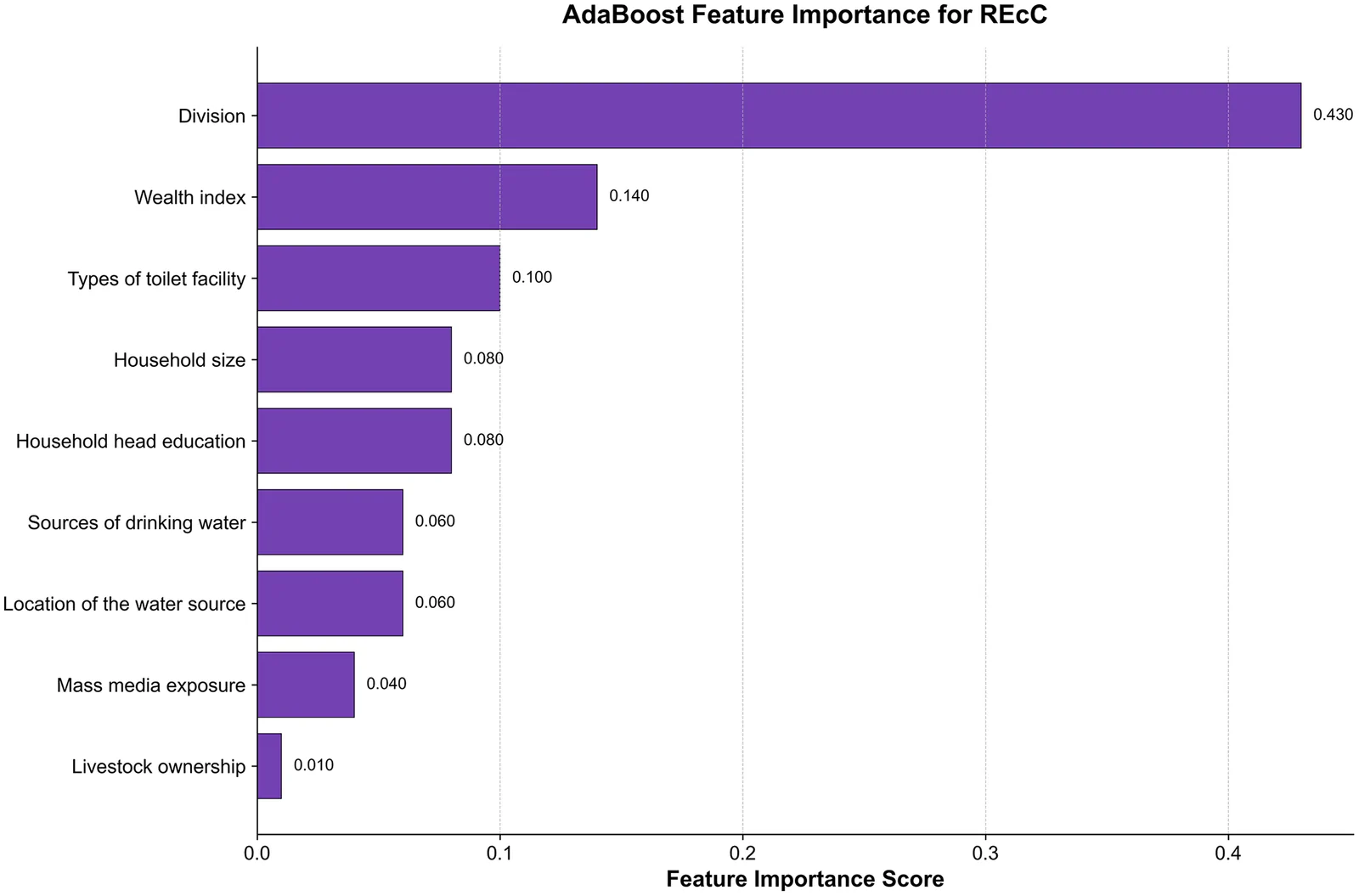
Feature importance based on AdaBoost.

Fig 7 illustrates the feature importance based on the AdaBoost model, providing valuable insights into the factors influencing *E. coli* levels in drinking water in Bangladesh. The analysis identifies five key variables division, wealth index, types of toilet facility, household size, and household head education as significant predictors of *E. coli* contamination in household water.

## Discussion

This cross-sectional study used 2019 MICS dataset and main purpose of this study is the assess the performance of ML algorithms to detect REcC in drinking water and identified the hotspot of highly contaminated district of risk of *E. coli* using the cluster dendrogram of the hierarchical clustering based on Wald’s criterion. To the best of our knowledge, there was no available study which have conducted in Bangladesh to focused on REcC based on the different ML classifiers and clustering approaches we have used in this study. A comparison table between our results and previous research is provided in S2 Table in S1 File.

This study also evaluated that prevalence of REcC in drinking water in Bangladesh was 39.2%. About 39% and 65% of pathogens were detected in point-of-drinking water samples and public domain water source samples, respectively, and classified as low-risk using PCR testing [58]. In a similar study researcher was done using API and conventional methods and concluded that the prevalence of *E. coli* from different water sources was 49.48% [59]. In our study REcC in drinking water in Bangladesh was significantly associated with several factors, including division, residence, household head education, household size, wealth index, livestock ownership, types of toilet facilities, sources of drinking water, treatment methods for drinking water [8], handwashing location, mass media exposure and maternal functional difficulties. Moreover, a univariate and multivariate analysis conducted in a study [4] which was found that demographic factors such as household heads, room occupancy rate and wealth status was significantly correlated with the occurrence of *E. coli* in stored drinking water [60].

We evaluated the performance of nine ML algorithms and one DL algorithm to predict REcC in drinking water across two scenarios: original (imbalanced) data and balanced data (SMOTE, with CV and hyperparameter tuning). The ML models included RF, LR, DT, GBA, AdaBoost, LightGBM, KNN, NB, and SVM; DL-MLP was used for DL. Across all ML and DL models, Scenario 2 (balanced data) outperformed Scenario 1 (imbalanced data). For Scenario 2, AdaBoost demonstrated the highest accuracy (81.6%) for correctly predicting REcC in drinking water, along with the highest recall (0.990) and F1-score (0.898). AdaBoost is often highly effective for classification because it combines weak learners to refine decision boundaries, capture feature interactions, and preserve good generalization, which frequently leads to superior accuracy and F1 scores compared with many other models [61,62]. From a practical perspective, our results suggest that boosting and other tree-based ensemble models (AdaBoost, GBA, and LightGBM) are strong first-choice methods for similar survey-based contamination studies, where predictors are largely categorical and relationships may be non-linear. In this dataset, LR showed weaker discrimination (lower AUC) than the ensemble approaches, which may reflect the importance of non-linear effects and interactions across household, environmental, and geographic factors.

Additionally, in terms of estimated AUC values, both the AdaBoost and GBA models outperformed LR, RF, GBA, DT, KNN, LightGBM, SVM, NB and DL-MLP models in predicting the REcC among households in Bangladesh. AdaBoost is versatile because it can use different types of weak learners (for example, decision stumps, SVMs, or neural networks) and be tuned to specific problems, which often improves its performance [63,64]. In the Milwaukee River system, physicochemical water-quality variables explained only a limited proportion of the variation in *E. coli* (R² = 0.29–0.42). The hybrid ANN–GBM model performed best, but prediction accuracy remained low (about 42%) [65]. RF achieved the highest R² value (0.933) when combining water quality and RGB data in the USA [66], as well as in predicting *E. coli* concentrations in the Marne River (Paris Area, France) [67]. KNN was the most accurate model for predicting *E. coli* concentrations in Midmar Dam, with XGBoost and SVM performing next best, while RF and ANN had the largest errors [68].

Moreover, RF has been more effective in predicting *E. coli* concentrations in agricultural pond waters, as it provided the smallest RMSE in almost all cases [17]. Furthermore, both RF and TPOT exhibited the best performance in predicting *E. coli* concentrations in the Göta älv river in Gothenburg, Sweden [69], and in Ethiopia [70]. On the other hand, SVM outperforms LR in 26 cities with an accuracy of 70%, except in Kathmandu, where it achieves 79%. In contrast, LR demonstrates a significantly lower accuracy of 61% across all cities [71].

For feature importance in the AdaBoost model, division was the strongest predictor of REcC in drinking water, followed by wealth index, types of toilet facility, household size, household head education, and source of drinking water. Recent study showed that climate, land use, and population density were key predictors of *E. coli* contamination in shallow tubewell water in Bangladesh [72]. A recent study using a Bayesian censored model on a similar dataset found that household *E. coli* levels were significantly associated with division, water source/location, treatment, handwashing, toilet type, education and wealth, and livestock ownership [73].

Despite all efforts to optimize ML algorithms even after class balancing for predicting REcC using the 2019 MICS dataset, models achieved a maximum accuracy of 81.6% but only a modest AUC of 0.686. This discrepancy reveals ML’s limited discriminatory ability to reliably predict contamination in household drinking water, even at moderate prevalence levels.

Furthermore, a study conducted in Ireland explored spatial mapping of *E. coli* occurrence in the community [74] to examine local geographic patterns of *E. coli* contamination. This study identified hotspot areas with high levels of contamination by using a cluster dendrogram from hierarchical clustering based on Wald’s criterion. In our study, we concluded that the highest REcC in household drinking water was observed in districts such as Bandarban, Dhaka, Habiganj, Narsingdi, Feni, Rangamati, Tangail, Rajbari, Lakshmipur, and Netrokona. These districts were marked as the first cluster, where more than 58% of cases were identified, with red spots indicating the highest levels of contamination. In contrast, districts represented in sky blue were grouped into the fourth cluster, which was characterized as low risk for *E. coli* contamination in household drinking water across Bangladesh.

This study has limitations. In terms of limitations, it is a cross-sectional study, so no cause-and-effect relationships can be established. Additionally, the analysis relied on socio-demographic variables available in the MICS dataset, excluding environmental factors such as distance from sewage treatment plants and population density because these variables were absent from the dataset and could impact contamination risks.

## Conclusion

In this study, we applied nine ML classifiers and one DL-MLP classifier to predict the REcC in household drinking water in Bangladesh using the 2019 MICS dataset. We also used agglomerative hierarchical clustering with Ward’s linkage to identify district-level hotspots. More than one-third of households were classified as having increased REcC in their drinking water. We compared model performance using accuracy, precision, recall, F1-score, Cohen’s kappa, ROC-AUC, and violin plots to summarize the distribution of performance across models. Across all models, SMOTE combined with CV and hyperparameter tuning substantially improved performance compared to the original imbalanced data. Based on ROC-AUC, the models showed limited discrimination between contaminated and non-contaminated household drinking-water samples, even though the best-performing model achieved 81.6% accuracy. Feature-importance results indicated that division, wealth index, type of toilet facility, household size, education, and source of drinking water were the most influential predictors of REcC. Future work should focus on improving predictive performance by incorporating additional environmental and spatial exposure variables where possible and by validating models on independent data.

## Supporting information

S1 File Supporting information figures and tables (S1-S6 Figs; S1-S2 Tables). (DOCX)
