# Supplementary material for: Use of machine learning to detect Escherichia coli in drinking water in Bangladesh
Source: PLoS One. 2026 Aug 20;21(8):e0343606. doi: 10.1371/journal.pone.0343606 (PMC13492749; doi:10.1371/journal.pone.0343606)
Supplement: Supplementary file 1 [file pone.0343606.s004.pdf]

The following is a point-by-point response to the editor and list of proposed changes:

EDITOR: PONE-D-25-52920R1
Use of machine learning to detect *Escherichia coli* in drinking water in Bangladesh
PLOS One

Dear Dr. Nobrega,

Thank you for submitting your manuscript to PLOS ONE. After careful consideration, we feel that it has merit but does not fully meet PLOS ONE's publication criteria as it currently stands. Therefore, we invite you to submit a revised version of the manuscript that addresses the points raised during the review process.

==============================
As discussed via email, we have rescinded the acceptance decision and returned the manuscript to you, so that you can make any necessary changes to the text and figures. Specifically, please add your updates to figure 3, figure 7, and the text of the manuscript. Once you resubmit your revised manuscript, it will be reviewed by the Academic Editor.
==============================

RE: Thank you. During proofreading, we identified inconsistencies between the methods text and figures. In the version that went to proofreading, feature selection described in the Results section (lines 205-209) mentioned fourteen variables, while the former SHapley Additive exPlanations (SHAP) summary plot (previous version of Figure 3) and underlying analyses were carried out using thirteen variables. The reason for discrepancy was the variable *Women functional difficulty*, which was missing from the original analysis. This required us to re-run the SHAP analysis with the extra variable and redraft Figure 3 to ensure full alignment with the methodology proposed for feature selection. Of note, *Women functional difficulty* had the lowest importance for predicting the outcome of interest and therefore excluded from further downstream analysis. We have now corrected Figure 3 to include all 14 features (updated Figure 3, lines 404, 408-409).

Figure 7 was also revised. In the original analysis underlying Figure 7, the variable *treatment of drinking water* was incorrectly forced into the model and consequently included in the original version of Figure 7. However, according to the proposed feature-selection methodology, this variable should not have been introduced in this analysis. Figure 7 has therefore been updated to exclude *treatment of drinking water*. While overall conclusions of the study remained unchanged (e.g., limited discriminatory power of machine learning to discriminate between contaminated and non-contaminated household drinking-water), this update required us to update the corresponding text in the results (lines 489-490), discussion (line 557) and conclusion (line 598).

We attached the 2 versions of Figures 3 and 7 to the submission package. These are labelled as Figure 3 (accepted) and Figure 3 (updated), and Figure 7 (accepted) and Figure 7 (updated). We propose to use the updated versions for the manuscript.

Additionally, we are proposing a list of minor edits to improve clarity. These are discussed below:

- In the Abstract section, we revised the text on page 2 (lines 48, 54, and 60) to improve readability.
- In the Introduction section, we clarified the study objectives on page 5 (lines 154 to 161).
- In the Outcome variable section, we revised “output variable” to “outcome variable” on line 188, corrected the unit from “ml” to “mL,” and provided the full form of CFU on page 6 (lines 191 to 200).
- In the Independent variables section, we revised “maternal functional difficulty” to the correct variable name, “women functional difficulty,” on page 6 (line 208).
- On line 226, we changed “chi-square” to “Chi-square.”
- Following PLOS ONE guidelines, we revised all subheadings into sentence case.
- In the machine learning classifiers section, we revised the text on page 8 (lines 256 to 259) to improve readability.
- In the Data preparation and model evaluation section, we italicized “Pipeline” on page 8 (line 270).
- In the Hyperparameter section, we italicized “*best_estimator*” on page 9 (line 309).
- For SMOTE (line 312), we had already provided the full form earlier in the manuscript; therefore, the short form was retained. We also made a minor revision on line 315 for clarity.
- On line 344, we revised the variable name to “women functional difficulties.”
- On line 432, we retained the short form for risk of *E. coli* contamination (REcC) as the acronym was already defined.
- On line 479, we used the short form for cross-validation (CV) given that the acronym was already defined.
- On lines 519 and 521, we revised the text to improve readability.
- On line 543, we retained the short form KNN because the full form had already been provided earlier.
- On line 549, we revised the text to improve readability.
