# Supplementary material for: Use of machine learning to detect Escherichia coli in drinking water in Bangladesh
Source: PLoS One. 2026 Aug 20;21(8):e0343606. doi: 10.1371/journal.pone.0343606 (PMC13492749; doi:10.1371/journal.pone.0343606)
Supplement: Supplementary file 2 [file pone.0343606.s001.docx]

**S1 File. Supporting information figures and tables**

*This file contains S1-S6 Figs and S1-S2 Tables.*

 **S1 Fig.** *The cluster dendrogram of the hierarchical clustering based on the Ward’s criterion results of the 64 districts with the risk of E. coli contamination among household drinking water in Bangladesh. The height of the branches indicates the dissimilarity between clusters. Here, y-axis representing the dissimilarity or distance between districts, and the x-axis listing the 64 districts of Bangladesh. A greater distance on the y-axis indicates a lower similarity between the districts.*

**S2 Fig.** *Correlations Among Categorical Features Using Cramer’s V Correlation Coefficient. Cramer’s V transforms the chi-square statistic onto a 0-1 scale, providing a straightforward and interpretable measure of association for categorical variables. In the lower panels, black indicates correlations among categorical independent features that tend toward negative (downward) associations. In the upper right panels, light pink represents positive (upward) correlations among categorical features. The white color highlights moderate associations, particularly between the place of handwashing and the location of water sources.*

**S3 Fig.** Distribution of Household Drinking-Water Source by Administrative Division.

**S4 Fig.** ROC curve for all models without SMOTE, the results from 10-fold cross-validation with hyperparameter tuning for all selected models

**S5 Fig.** Classification performance measure of the algorithms and comparison for imbalanced (original data).

**S6 Fig.** *Radar Plot Comparing the Performance of Various Machine Learning Models—Adaptive Boosting (AdaBoost), Deep Learning (DL-MLP), Decision Trees (DT), Gradient Boosting Algorithm (GBA), K-Nearest Neighbor (KNN), Light Gradient-Boosting Machine (LightGBM), Logistic Regression (LR), Naïve Bayes (NB), Random Forest (RF), and Support Vector Machine (SVM) in predicting the risk of E. coli contamination in household drinking water in Bangladesh. The performance evaluation metrics include accuracy, precision, Cohen's kappa, recall, and F1 score. Each machine learning method is represented on a separate axis, scaled from 0 to 80% for each metric, with all axes converging at the center of the chart. Radar plots are particularly useful for highlighting outliers and identifying areas of overlap, effectively demonstrating that the AdaBoost classifier slightly outperforms the others across multiple evaluation metrics.*

**S1 Table.** Hyperparameter tuning details (GridSearchCV) for all algorithms

| **Algorithm** | **Hyperparameters optimized** | **Values tested (grid)** | **Validation and selection criterion** |
| --- | --- | --- | --- |
| LR | Regularization strength (C); class_weight; solver | C: 0.001, 0.01, 0.1, 1, 10, 100; class_weight: None, balanced; solver: lbfgs | Grid search using stratified 10-fold CV on the training set; selected the setting with the highest mean F1-score (positive class) across folds. SMOTE applied within each training fold only. |
| NB | var_smoothing | 1e−12, 1e−11, 1e−10, 1e−9, 1e−8, 1e−7 | Stratified 10-fold CV; best mean F1-score selected. SMOTE within-fold only. |
| DT | max_depth; min_samples_split; min_samples_leaf; class_weight | max_depth: None, 5, 10, 15, 20; min_samples_split: 2, 5, 10, 20; min_samples_leaf: 1, 2, 4, 8; class_weight: None, balanced | Stratified 10-fold CV; best mean F1-score selected. SMOTE within-fold only. |
| RF | n_estimators; max_depth; min_samples_split; min_samples_leaf; max_features; class_weight | n_estimators: 100, 200, 300; max_depth: None, 10, 20, 30; min_samples_split: 2, 5, 10; min_samples_leaf: 1, 2, 4; max_features: sqrt, log2; class_weight: None, balanced | Stratified 10-fold CV; best mean F1-score selected. SMOTE within-fold only. |
| SVM | C; kernel; gamma; class_weight | C: 0.1, 1, 10, 100; kernel: linear, rbf; gamma: scale, auto; class_weight: None, balanced | Stratified 10-fold CV; best mean F1-score selected. SMOTE within-fold only. |
| KNN | n_neighbors; weights; p (distance metric) | n_neighbors: 3, 5, 7, 9, 11; weights: uniform, distance; p: 1 (Manhattan), 2 (Euclidean) | Stratified 10-fold CV; best mean F1-score selected. SMOTE within-fold only. |
| AdaBoost | n_estimators; learning_rate | n_estimators: 50, 100, 200; learning_rate: 0.01, 0.1, 0.5, 1.0 | Stratified 10-fold CV; best mean F1-score selected. SMOTE within-fold only. |
| GBA | n_estimators; learning_rate; max_depth; subsample | n_estimators: 100, 200, 300; learning_rate: 0.01, 0.1, 0.2; max_depth: 3, 4, 5; subsample: 0.8, 1.0 | Stratified 10-fold CV; best mean F1-score selected. SMOTE within-fold only. |
| LightGBM | n_estimators; learning_rate; num_leaves; max_depth; min_child_samples | n_estimators: 100, 200, 300; learning_rate: 0.01, 0.05, 0.1; num_leaves: 31, 63; max_depth: 5, 7, −1; min_child_samples: 10, 20 | Stratified 10-fold CV; best mean F1-score selected. SMOTE within-fold only. |
| DL-MLP | hidden_layer_sizes; activation; L2 penalty (alpha); learning_rate_init; early_stopping | hidden_layer_sizes: (64), (128), (64, 32); activation: relu, tanh; alpha: 0.0001, 0.001, 0.01; learning_rate_init: 0.001, 0.01; early_stopping: True | Stratified 10-fold CV; best mean F1-score selected. SMOTE within-fold only. Early stopping used an internal split within each training fold. |

**S2 Table.** Comparison aligned with the Discussion (current study vs previous research)

| Current study (Bangladesh; MICS 2019) | Previous research (what has been reported) |
| --- | --- |
| Study design and scope: Cross-sectional analysis of the nationally representative 2019 MICS dataset to predict REcC and identify hotspot districts | Most studies are either (i) laboratory-based sampling from selected water sources, (ii) regression-based risk-factor studies, or (iii) environmental monitoring models; few use nationally representative survey predictors and district-level hotspot clustering together |
| Novelty/contribution: Benchmarked 9 ML classifiers + 1 DL model under two scenarios (imbalanced vs SMOTE-balanced with CV and tuning) and applied hierarchical clustering (Wald’s criterion) to classify districts into risk clusters | To our knowledge, no Bangladesh study has combined multi-model ML benchmarking for REcC with dendrogram-based district clustering using a survey dataset like MICS |
| Prevalence: REcC prevalence in drinking water was 39.2% | Comparable prevalence levels reported in other sampling frames: 39% point-of-drinking water and 65% public domain sources by PCR (Saima et al., 2023); 49.48% from different water sources using API/conventional methods (Odonkor & Addo, 2018) |
| Determinants (association findings): REcC associated with division, residence, household head education, household size, wealth index, livestock ownership, toilet type, water source, water treatment, handwashing location, mass media exposure, maternal functional difficulties | Similar themes recur: WASH and water source/treatment factors (Dada et al., 2013) and sociodemographic factors such as household head characteristics, crowding/room occupancy, and wealth linked with E. coli occurrence (Fong et al., 2007; Vannavong et al., 2018) |
| Modeling strategy: Compared performance across Scenario 1 (imbalanced) and Scenario 2 (SMOTE + 10-fold CV + hyperparameter tuning) | Many prior studies do not use a common ML benchmarking framework (same dataset, same CV scheme, same metric set); performance reporting is often not directly comparable across studies |
| Best classifier (Scenario 2): AdaBoost achieved the highest accuracy (81.6%), recall (0.990), and F1-score (0.898) | AdaBoost is often effective in classification because boosting can capture non-linearities and interactions and refine decision boundaries (Martinez & Gray, 2016; Maji et al., 2024); related work also notes its flexibility via choice of weak learners and tuning (Gai et al., 2016; Nabi et al., 2023) |
| Discrimination (AUC): AUC values were modest overall; AdaBoost AUC = 0.686 (highest), followed closely by GBA AUC = 0.685; LR lowest (0.591) | Environmental and multi-source studies show wide variability depending on predictors/outcome: ANN–GBM reached ~42% accuracy with physicochemical predictors in a river system (Nafsin & Li, 2023); RF achieved high R² = 0.933 when combining water-quality and RGB data (Hong et al., 2024) and performed well in river concentration prediction (Naloufi et al., 2021); kNN best in a dam study (Ibrahim et al., 2025); RF strong in ponds and other river systems (Stocker et al., 2022; Sokolova et al., 2022; Ambel et al., 2023); SVM often outperformed LR in multi-city prediction (Kuroki et al., 2023) |
| Interpretation of performance: Despite class balancing and tuning, the best model achieved high accuracy but only modest AUC (<0.70), suggesting limited discriminatory signal in MICS predictors for individual household prediction | Prior work similarly suggests that prediction performance is constrained when key microbial/environmental drivers are not directly measured; stronger results are often reported when richer water-quality, climatic, land-use, or sensor predictors are available |
| Feature importance (AdaBoost): Division strongest predictor, followed by wealth index, household head education, and drinking-water source | Bangladesh-related findings align partly but use different predictor sets or modeling frameworks: climate, land use, and population density were key predictors for shallow tubewell contamination (Wu et al., 2025); Bayesian censored modeling found division, water source/location, treatment, handwashing, toilet type, education/wealth, and livestock ownership associated with household E. coli (Haq et al., 2025) |
| Hotspot detection: Hierarchical clustering identified a high-risk cluster including Bandarban, Dhaka, Habiganj, Narsingdi, Feni, Rangamati, Tangail, Rajbari, Lakshmipur, and Netrokona; first cluster contained >58% of cases (high-risk “red spots”) | Spatial mapping and clustering have been used to detect geographic hotspots of E. coli occurrence in other settings (e.g., Ireland) using dendrogram-based hierarchical clustering (Samarasundera et al., 2012) |
| Practical implication: For survey-based contamination risk prediction with mostly categorical predictors, boosting/tree ensembles (AdaBoost, GBA, LightGBM, RF) are reasonable first-choice models; however, modest AUC indicates limits for sensitive household-level screening using MICS predictors alone | Prior studies support that the “best” model depends on data type: ensembles often perform well, but gains are larger when predictors include direct water-quality, environmental, or sensor information rather than survey proxies |
