# Supplementary material for: Use of machine learning to detect Escherichia coli in drinking water in Bangladesh
Source: PLoS One. 2026 Aug 20;21(8):e0343606. doi: 10.1371/journal.pone.0343606 (PMC13492749; doi:10.1371/journal.pone.0343606)
Supplement: Supplementary file 3 [file pone.0343606.s003.docx]

**Rebuttal Letter**

**Date: January 11, 2026**

Dear Dr. Saif,

On behalf of all the authors, I wish to convey our gratitude for the critical and constructive feedback that we received from reviewers in our recent submission (Manuscript number: PONE-D-25-52920) entitled “**Use of machine learning to detect *Escherichia coli* in drinking water in Bangladesh***.*” The manuscript has been revised in accordance with the feedback provided. All changes are highlighted using track changes in the revised manuscript. I hope our efforts satisfy the requirements of the journal. I will be looking forward to your positive response. Thank you for your time and consideration.

Iqramul Haq (on the behalf of authors)

Corresponding author

**##** **The following is a point-by-point response to the reviewer(s) comments:**

**Reviewer #1:** The paper presents an interesting application of machine learning to the selected dataset. The topic is relevant and timely, and the manuscript is generally well organized. However, several methodological and reporting aspects require clarification or further development to enhance the rigor, interpretability, and overall contribution of the study.
**Author Response:** Thank you for your comments. We have revised the manuscript following your suggestions.

**Literature Review:**

The introduction provides a useful overview of related work, but it would benefit from including the accuracy or performance metrics reported in previous studies. This would help contextualize the authors’ results and allow for direct comparison of findings. Additionally, if any machine learning analyses have been previously conducted on the same dataset, these should be reviewed and referenced. The authors could then discuss their results in comparison with those prior findings in the discussion section.

**Author Response:** We have updated the Introduction section. To the best of our knowledge, no previous study has used a machine-learning approach with this dataset, but alternative approaches were indeed used to analyze the same dataset. We added that to the introduction (pages 4-5).

**Use of SHAP and Feature Engineering:**
The paper correctly highlights the use of SHAP for model interpretability. However, it would be beneficial to explain how SHAP can be leveraged to improve model performance, not just interpretation. Similarly, since the paper mentions feature engineering, the authors could explore how SHAP and feature engineering together might be used to exclude unimportant features, which could potentially improve model accuracy or stability.

**Author Response:** Thank you for your feedback. We have incorporated this information into the SHAP subsection within the Statistical Analysis section (pages 7-8).

**Use of SMOTE:**
The authors apply SMOTE but do not clearly explain why it was required. The manuscript should describe the class imbalance in the dataset and justify the need for oversampling. Providing before-and-after class distributions or comparing model performance with and without SMOTE would make this methodological choice more transparent.

**Author Response:** We have made the necessary changes to the manuscript. We also evaluated model performance metrics before and after SMOTE in the revised version (with SMOTE: pages 10, and 16-19, without SMOTE: see the supplemental pages 3-4).

**Hyperparameter Tuning:**
The manuscript mentions the use of Grid Search for hyperparameter tuning but does not provide sufficient detail about the process. The authors should specify which parameters were optimized, the ranges or values tested, and how the tuning process was validated (for example, cross-validation approach or performance metric used for selection). It would also strengthen the study to include performance metrics before and after tuning to show the effect of this optimization.

**Author Response:** Thank you for your comment. We addressed this in the Hyperparameter Tuning subsection of the Statistical Analysis section (page10). We also compared model performance before and after hyperparameter tuning (with hyperparameter tuning: pages 16-19, without hyperparameter tuning: see the supplemental pages 3-4)

**Variable Analysis and Visualization:**
Table 2 presents variable distributions and percentages, which is informative. However, this section could be enhanced by exploring interactions between key variables. For example, examining the relationship between “Division” and “Source of Drinking Water”—identified as important features by SHAP—could reveal deeper insights. Adding visualizations such as heatmaps or grouped bar charts based on two-variable analyses would add another dimension to the findings and improve interpretability.

**Author Response:** We have revised and added this information to the Cramer's V correlation subsection in the Results section (page 16).

**Figure Captions:**
Several figure captions are overly long. It would be preferable to move part of the explanation to the main text, keeping the captions concise and focused on describing what the figures show rather than providing detailed interpretations.

**Author Response:** That has now been adjusted for all figures. Thank you.

**Model Performance and Discussion:**

The authors report multiple performance metrics, including F1-score, precision, recall, and AUC, which is commendable. However, the discussion should further address whether the achieved accuracy (approximately 0.7) is acceptable or competitive within their specific research field. Comparing this performance to similar studies would provide important context for evaluating the model’s effectiveness and practical applicability.

**Author Response:** We have revised the Discussion section accordingly (pages 21-22). Note that, after models were revisited following suggestions from reviewers, model performance improved slightly after applying SMOTE, 10-fold cross-validation, and hyperparameter tuning.

**Reviewer #2**: The manuscript presents an important application of classical and deep learning models for predicting E. coli contamination in household drinking water in Bangladesh using a nationally representative dataset.

Two Minor, Yet Scientifically Important Improvements
1. Clarify the rationale behind SMOTE for an already balanced dataset segment

- The reported prevalence of contamination is ~39% vs 61%. This is moderately imbalanced, not severely.

**Author Response:** Thank you. The outcome was indeed moderately imbalanced. Our rationale for using SMOTE was to test its ability to increase accuracy, especially for the contaminated subset. We added the rationale for SMOTE in page 10. Note that we now compare this approach with the original imbalanced dataset (with SMOTE: pages 10, and 16-19, without SMOTE: see the supplemental pages 3-4).

- Please briefly justify the use of SMOTE and discuss whether oversampling may have artificially inflated model performance or introduced bias, especially since several models showed modest AUC values (<0.70).

**Author Response:** As explained above, we now compare the SMOTE approach with the original imbalanced dataset (with SMOTE: pages 10, and 16-19, without SMOTE: see the supplemental pages 3-4). Additionally, please note that SMOTE was used only in the training dataset -the validation folds and the final held-out test set remained non-synthetic.


2. Add a short explanation of why AdaBoost outperformed other models

- AdaBoost achieved the highest accuracy and F1 score, but the manuscript does not interpret why it may be more suitable for this problem (e.g., handling weak learners, sensitivity to decision boundaries, feature interactions).
**Author Response:** This has now been added to the Discussion section (page 21).

- A 2–3 sentence interpretation would strengthen the Discussion and provide insight for practitioners choosing ML techniques for similar epidemiological datasets.

**Author Response:** This has now been added to the Discussion section (page 21).

**Reviewer #3:** This research represents an important scientific contribution in the field of public health and drinking water quality .This is achieved by applying advanced machine learning algorithms to nationally representative population data, a successful experiment in Bangladesh for predicting the risk of drinking water contamination ,but i have Nom. of comments
1-Some paragraphs are repetitive and need academic rewriting.

**Author Response:** Thank you for your comments. We have the entire manuscript to the best of our ability.

2-Add a comparison table between your results and the results of previous research.

**Author Response:** Thank you. That has now been added to supplemental files (Table 2).

3-Weaknesses in the wording of research contributions. What's new?

**Author Response:** We have added this information to the last paragraph of the Introduction section (page 5).

4- The statistical performance of the models was weak, and I suggest to improve by adding environmental variables related to potential pollution, such as:
• Distance from sewage treatment plants
• Population density

**Author Response:** We agree that the performance was weak and that is discussed in the last paragraph of the Discussion section (page 23). Additionally, please note that we now have optimized our hyperparameter turning approach based on reviewers feedback, which has increased our overall accuracy from 70.2% to 81.6% (pages 16-19).

5- The accuracy of the results is important, and this observation is crucial. These values are considered very weak for sensitive health applications. Had the researcher used appropriate deep learning algorithms for their data, the results would have been better, as these are important findings.

**Author Response:** Author Response: Thank you for this observation. We agree that the reported performance is insufficient for sensitive, individual-level health decision-making, and we have revised the manuscript to clarify that these models are not intended for clinical use. We included a commonly used deep learning multilayer perceptron (DL-MLP), but its performance was not substantially better than the classical ML models (page 16-19. Additionally, we now have optimized our hyperparameter turning approach based on reviewers feedback, which has increased our overall accuracy from 70.2% to 81.6% (pages 16-19).

**Reviewer #4:** The research was good in terms of the sources and samples used, but the practical aspect was not clear enough. The practical side needs further explanation; there should be a brief explanation of the functions used to classify the samples, as well as an explanation of how the system used in the sample analysis was evaluated.

**Author Response:** Thank you for your comments. We added a paragraph discussing the practical aspects of the study. Additionally, we have revised the Methods section accordingly to include the functions used (pages 8-10).
