# Supplementary material for: Use of machine learning to detect Escherichia coli in drinking water in Bangladesh
Source: PLoS One. 2026 Aug 20;21(8):e0343606. doi: 10.1371/journal.pone.0343606 (PMC13492749; doi:10.1371/journal.pone.0343606)
Supplement: Peer review history

# Peer review: Decision Letter 0

**DOI:** 10.1371/journal.pone.0343606.r001

**Authors:** Sohail Saif

1 Dec 2025

PONE-D-25-52920

Use of machine learning to detect Escherichia coli in drinking water in Bangladesh

PLOS ONE

Dear Dr. Haq,

Thank you for submitting your manuscript to PLOS ONE. After careful consideration, we feel that it has merit but does not fully meet PLOS ONE’s publication criteria as it currently stands. Therefore, we invite you to submit a revised version of the manuscript that addresses the points raised during the review process.

Please submit your revised manuscript by Jan 15 2026 11:59PM. If you will need more time than this to complete your revisions, please reply to this message or contact the journal office at plosone@plos.org. When you're ready to submit your revision, log on to https://www.editorialmanager.com/pone/ and select the 'Submissions Needing Revision' folder to locate your manuscript file.

Please include the following items when submitting your revised manuscript:

- A rebuttal letter that responds to each point raised by the academic editor and reviewer(s). You should upload this letter as a separate file labeled 'Response to Reviewers'.
- A marked-up copy of your manuscript that highlights changes made to the original version. You should upload this as a separate file labeled 'Revised Manuscript with Track Changes'.
- An unmarked version of your revised paper without tracked changes. You should upload this as a separate file labeled 'Manuscript'.

If you would like to make changes to your financial disclosure, please include your updated statement in your cover letter. Guidelines for resubmitting your figure files are available below the reviewer comments at the end of this letter.

If applicable, we recommend that you deposit your laboratory protocols in protocols.io to enhance the reproducibility of your results. Protocols.io assigns your protocol its own identifier (DOI) so that it can be cited independently in the future. For instructions see: https://journals.plos.org/plosone/s/submission-guidelines#loc-laboratory-protocols. Additionally, PLOS ONE offers an option for publishing peer-reviewed Lab Protocol articles, which describe protocols hosted on protocols.io. Read more information on sharing protocols at https://plos.org/protocols?utm_medium=editorial-email&utm_source=authorletters&utm_campaign=protocols.

We look forward to receiving your revised manuscript.

Kind regards,

Sohail Saif, Ph.D

Academic Editor

PLOS ONE

**Journal Requirements:**

When submitting your revision, we need you to address these additional requirements.

1. Please ensure that your manuscript meets PLOS ONE's style requirements, including those for file naming. The PLOS ONE style templates can be found at

https://journals.plos.org/plosone/s/file?id=wjVg/PLOSOne_formatting_sample_main_body.pdf and

https://journals.plos.org/plosone/s/file?id=ba62/PLOSOne_formatting_sample_title_authors_affiliations.pdf

2. Please note that PLOS One has specific guidelines on code sharing for submissions in which author-generated code underpins the findings in the manuscript. In these cases, we expect all author-generated code to be made available without restrictions upon publication of the work. Please review our guidelines at https://journals.plos.org/plosone/s/materials-and-software-sharing#loc-sharing-code and ensure that your code is shared in a way that follows best practice and facilitates reproducibility and reuse.

3. PLOS requires an ORCID iD for the corresponding author in Editorial Manager on papers submitted after December 6th, 2016. Please ensure that you have an ORCID iD and that it is validated in Editorial Manager. To do this, go to ‘Update my Information’ (in the upper left-hand corner of the main menu), and click on the Fetch/Validate link next to the ORCID field. This will take you to the ORCID site and allow you to create a new iD or authenticate a pre-existing iD in Editorial Manager.

4. We note that Figure 2 in your submission contain map images which may be copyrighted. All PLOS content is published under the Creative Commons Attribution License (CC BY 4.0), which means that the manuscript, images, and Supporting Information files will be freely available online, and any third party is permitted to access, download, copy, distribute, and use these materials in any way, even commercially, with proper attribution. For these reasons, we cannot publish previously copyrighted maps or satellite images created using proprietary data, such as Google software (Google Maps, Street View, and Earth). For more information, see our copyright guidelines: http://journals.plos.org/plosone/s/licenses-and-copyright.

We require you to either (1) present written permission from the copyright holder to publish these figures specifically under the CC BY 4.0 license, or (2) remove the figures from your submission:

A.  You may seek permission from the original copyright holder of Figures 1 and 2 to publish the content specifically under the CC BY 4.0 license.

We recommend that you contact the original copyright holder with the Content Permission Form (http://journals.plos.org/plosone/s/file?id=7c09/content-permission-form.pdf) and the following text:

“I request permission for the open-access journal PLOS ONE to publish XXX under the Creative Commons Attribution License (CCAL) CC BY 4.0 (http://creativecommons.org/licenses/by/4.0/). Please be aware that this license allows unrestricted use and distribution, even commercially, by third parties. Please reply and provide explicit written permission to publish XXX under a CC BY license and complete the attached form.”

Please upload the completed Content Permission Form or other proof of granted permissions as an "Other" file with your submission.

In the figure caption of the copyrighted figure, please include the following text: “Reprinted from [ref] under a CC BY license, with permission from [name of publisher], original copyright [original copyright year].”

B. If you are unable to obtain permission from the original copyright holder to publish these figures under the CC BY 4.0 license or if the copyright holder’s requirements are incompatible with the CC BY 4.0 license, please either i) remove the figure or ii) supply a replacement figure that complies with the CC BY 4.0 license. Please check copyright information on all replacement figures and update the figure caption with source information. If applicable, please specify in the figure caption text when a figure is similar but not identical to the original image and is therefore for illustrative purposes only.

The following resources for replacing copyrighted map figures may be helpful:

USGS National Map Viewer (public domain): http://viewer.nationalmap.gov/viewer/

The Gateway to Astronaut Photography of Earth (public domain): http://eol.jsc.nasa.gov/sseop/clickmap/

Maps at the CIA (public domain): https://www.cia.gov/library/publications/the-world-factbook/index.html and https://www.cia.gov/library/publications/cia-maps-publications/index.html

NASA Earth Observatory (public domain): http://earthobservatory.nasa.gov/

Landsat: http://landsat.visibleearth.nasa.gov/

USGS EROS (Earth Resources Observatory and Science (EROS) Center) (public domain): http://eros.usgs.gov/#

Natural Earth (public domain): http://www.naturalearthdata.com/

5. Please include captions for your Supporting Information files at the end of your manuscript, and update any in-text citations to match accordingly. Please see our Supporting Information guidelines for more information: http://journals.plos.org/plosone/s/supporting-information.

6. If the reviewer comments include a recommendation to cite specific previously published works, please review and evaluate these publications to determine whether they are relevant and should be cited. There is no requirement to cite these works unless the editor has indicated otherwise.

[Note: HTML markup is below. Please do not edit.]

Reviewers' comments:

Reviewer's Responses to Questions

**Comments to the Author**

1. Is the manuscript technically sound, and do the data support the conclusions?

The manuscript must describe a technically sound piece of scientific research with data that supports the conclusions. Experiments must have been conducted rigorously, with appropriate controls, replication, and sample sizes. The conclusions must be drawn appropriately based on the data presented.

Reviewer #1: Yes

Reviewer #2: Yes

Reviewer #3: Yes

Reviewer #4: Yes

**********

2. Has the statistical analysis been performed appropriately and rigorously?

Reviewer #1: Yes

Reviewer #2: Yes

Reviewer #3: No

Reviewer #4: Yes

**********

3. Have the authors made all data underlying the findings in their manuscript fully available?

The PLOS Data policy requires authors to make all data underlying the findings described in their manuscript fully available without restriction, with rare exception (please refer to the Data Availability Statement in the manuscript PDF file). The data should be provided as part of the manuscript or its supporting information, or deposited to a public repository. For example, in addition to summary statistics, the data points behind means, medians and variance measures should be available. If there are restrictions on publicly sharing data—e.g. participant privacy or use of data from a third party—those must be specified.

Reviewer #1: Yes

Reviewer #2: Yes

Reviewer #3: Yes

Reviewer #4: Yes

**********

4. Is the manuscript presented in an intelligible fashion and written in standard English?

PLOS ONE does not copyedit accepted manuscripts, so the language in submitted articles must be clear, correct, and unambiguous. Any typographical or grammatical errors should be corrected at revision, so please note any specific errors here.

Reviewer #1: Yes

Reviewer #2: Yes

Reviewer #3: No

Reviewer #4: Yes

**********

5. Review Comments to the Author

Please use the space provided to explain your answers to the questions above. You may also include additional comments for the author, including concerns about dual publication, research ethics, or publication ethics. (Please upload your review as an attachment if it exceeds 20,000 characters)

Reviewer #1: The paper presents an interesting application of machine learning to the selected dataset. The topic is relevant and timely, and the manuscript is generally well organized. However, several methodological and reporting aspects require clarification or further development to enhance the rigor, interpretability, and overall contribution of the study.

Literature Review:

The introduction provides a useful overview of related work, but it would benefit from including the accuracy or performance metrics reported in previous studies. This would help contextualize the authors’ results and allow for direct comparison of findings. Additionally, if any machine learning analyses have been previously conducted on the same dataset, these should be reviewed and referenced. The authors could then discuss their results in comparison with those prior findings in the discussion section.

Use of SHAP and Feature Engineering:

The paper correctly highlights the use of SHAP for model interpretability. However, it would be beneficial to explain how SHAP can be leveraged to improve model performance, not just interpretation. Similarly, since the paper mentions feature engineering, the authors could explore how SHAP and feature engineering together might be used to exclude unimportant features, which could potentially improve model accuracy or stability.

Use of SMOTE:

The authors apply SMOTE but do not clearly explain why it was required. The manuscript should describe the class imbalance in the dataset and justify the need for oversampling. Providing before-and-after class distributions or comparing model performance with and without SMOTE would make this methodological choice more transparent.

Hyperparameter Tuning:

The manuscript mentions the use of Grid Search for hyperparameter tuning but does not provide sufficient detail about the process. The authors should specify which parameters were optimized, the ranges or values tested, and how the tuning process was validated (for example, cross-validation approach or performance metric used for selection). It would also strengthen the study to include performance metrics before and after tuning to show the effect of this optimization.

Variable Analysis and Visualization:

Table 2 presents variable distributions and percentages, which is informative. However, this section could be enhanced by exploring interactions between key variables. For example, examining the relationship between “Division” and “Source of Drinking Water”—identified as important features by SHAP—could reveal deeper insights. Adding visualizations such as heatmaps or grouped bar charts based on two-variable analyses would add another dimension to the findings and improve interpretability.

Figure Captions:

Several figure captions are overly long. It would be preferable to move part of the explanation to the main text, keeping the captions concise and focused on describing what the figures show rather than providing detailed interpretations.

Model Performance and Discussion:

The authors report multiple performance metrics, including F1-score, precision, recall, and AUC, which is commendable. However, the discussion should further address whether the achieved accuracy (approximately 0.7) is acceptable or competitive within their specific research field. Comparing this performance to similar studies would provide important context for evaluating the model’s effectiveness and practical applicability.

Reviewer #2: The manuscript presents an important application of classical and deep learning models for predicting E. coli contamination in household drinking water in Bangladesh using a nationally representative dataset.

Two Minor, Yet Scientifically Important Improvements

1. Clarify the rationale behind SMOTE for an already balanced dataset segment

- The reported prevalence of contamination is ~39% vs 61%. This is moderately imbalanced, not severely.

- Please briefly justify the use of SMOTE and discuss whether oversampling may have artificially inflated model performance or introduced bias, especially since several models showed modest AUC values (<0.70).

2. Add a short explanation of why AdaBoost outperformed other models

- AdaBoost achieved the highest accuracy and F1 score, but the manuscript does not interpret why it may be more suitable for this problem (e.g., handling weak learners, sensitivity to decision boundaries, feature interactions).

- A 2–3 sentence interpretation would strengthen the Discussion and provide insight for practitioners choosing ML techniques for similar epidemiological datasets.

Reviewer #3: This research represents an important scientific contribution in the field of public health and drinking water quality .This is achieved by applying advanced machine learning algorithms to nationally representative population data, a successful experiment in Bangladesh for predicting the risk of drinking water contamination ,but i have Nom. of comments

1-Some paragraphs are repetitive and need academic rewriting.

2-Add a comparison table between your results and the results of previous research.

3-Weaknesses in the wording of research contributions. What's new?

4- The statistical performance of the models was weak, and I suggest to improve by adding environmental variables related to potential pollution, such as:

• Distance from sewage treatment plants

• Population density

5- The accuracy of the results is important, and this observation is crucial. These values are considered very weak for sensitive health applications. Had the researcher used appropriate deep learning algorithms for their data, the results would have been better, as these are important findings.

Reviewer #4: The research was good in terms of the sources and samples used, but the practical aspect was not clear enough. The practical side needs further explanation; there should be a brief explanation of the functions used to classify the samples, as well as an explanation of how the system used in the sample analysis was evaluated.

**********

6. PLOS authors have the option to publish the peer review history of their article (what does this mean?). If published, this will include your full peer review and any attached files.

If you choose “no”, your identity will remain anonymous but your review may still be made public.

**Do you want your identity to be public for this peer review?**  For information about this choice, including consent withdrawal, please see our Privacy Policy.

Reviewer #1: No

Reviewer #2: No

Reviewer #3: No

Reviewer #4: No

**********

[NOTE: If reviewer comments were submitted as an attachment file, they will be attached to this email and accessible via the submission site. Please log into your account, locate the manuscript record, and check for the action link "View Attachments". If this link does not appear, there are no attachment files.]

To ensure your figures meet our technical requirements, please review our figure guidelines: https://journals.plos.org/plosone/s/figures

You may also use PLOS’s free figure tool, NAAS, to help you prepare publication quality figures: https://journals.plos.org/plosone/s/figures#loc-tools-for-figure-preparation.

NAAS will assess whether your figures meet our technical requirements by comparing each figure against our figure specifications.

# Author comment: Author response to Decision Letter 1

**DOI:** 10.1371/journal.pone.0343606.r002

14 Jan 2026

Rebuttal Letter

Date: January 11, 2026

Dear Dr. Saif,

On behalf of all the authors, I wish to convey our gratitude for the critical and constructive feedback that we received from reviewers in our recent submission (Manuscript number: PONE-D-25-52920) entitled “Use of machine learning to detect Escherichia coli in drinking water in Bangladesh.” The manuscript has been revised in accordance with the feedback provided. All changes are highlighted using track changes in the revised manuscript. I hope our efforts satisfy the requirements of the journal. I will be looking forward to your positive response. Thank you for your time and consideration.

Iqramul Haq (on the behalf of authors)

Corresponding author

## The following is a point-by-point response to the reviewer(s) comments:

Reviewer #1: The paper presents an interesting application of machine learning to the selected dataset. The topic is relevant and timely, and the manuscript is generally well organized. However, several methodological and reporting aspects require clarification or further development to enhance the rigor, interpretability, and overall contribution of the study.

Author Response: Thank you for your comments. We have revised the manuscript following your suggestions.

Literature Review:

The introduction provides a useful overview of related work, but it would benefit from including the accuracy or performance metrics reported in previous studies. This would help contextualize the authors’ results and allow for direct comparison of findings. Additionally, if any machine learning analyses have been previously conducted on the same dataset, these should be reviewed and referenced. The authors could then discuss their results in comparison with those prior findings in the discussion section.

Author Response: We have updated the Introduction section. To the best of our knowledge, no previous study has used a machine-learning approach with this dataset, but alternative approaches were indeed used to analyze the same dataset. We added that to the introduction (pages 4-5).

Use of SHAP and Feature Engineering:

The paper correctly highlights the use of SHAP for model interpretability. However, it would be beneficial to explain how SHAP can be leveraged to improve model performance, not just interpretation. Similarly, since the paper mentions feature engineering, the authors could explore how SHAP and feature engineering together might be used to exclude unimportant features, which could potentially improve model accuracy or stability.

Author Response: Thank you for your feedback. We have incorporated this information into the SHAP subsection within the Statistical Analysis section (pages 7-8).

Use of SMOTE:

The authors apply SMOTE but do not clearly explain why it was required. The manuscript should describe the class imbalance in the dataset and justify the need for oversampling. Providing before-and-after class distributions or comparing model performance with and without SMOTE would make this methodological choice more transparent.

Author Response: We have made the necessary changes to the manuscript. We also evaluated model performance metrics before and after SMOTE in the revised version (with SMOTE: pages 10, and 16-19, without SMOTE: see the supplemental pages 3-4).

Hyperparameter Tuning:

The manuscript mentions the use of Grid Search for hyperparameter tuning but does not provide sufficient detail about the process. The authors should specify which parameters were optimized, the ranges or values tested, and how the tuning process was validated (for example, cross-validation approach or performance metric used for selection). It would also strengthen the study to include performance metrics before and after tuning to show the effect of this optimization.

Author Response: Thank you for your comment. We addressed this in the Hyperparameter Tuning subsection of the Statistical Analysis section (page10). We also compared model performance before and after hyperparameter tuning (with hyperparameter tuning: pages 16-19, without hyperparameter tuning: see the supplemental pages 3-4)

Variable Analysis and Visualization:

Table 2 presents variable distributions and percentages, which is informative. However, this section could be enhanced by exploring interactions between key variables. For example, examining the relationship between “Division” and “Source of Drinking Water”—identified as important features by SHAP—could reveal deeper insights. Adding visualizations such as heatmaps or grouped bar charts based on two-variable analyses would add another dimension to the findings and improve interpretability.

Author Response: We have revised and added this information to the Cramer's V correlation subsection in the Results section (page 16).

Figure Captions:

Several figure captions are overly long. It would be preferable to move part of the explanation to the main text, keeping the captions concise and focused on describing what the figures show rather than providing detailed interpretations.

Author Response: That has now been adjusted for all figures. Thank you.

Model Performance and Discussion:

The authors report multiple performance metrics, including F1-score, precision, recall, and AUC, which is commendable. However, the discussion should further address whether the achieved accuracy (approximately 0.7) is acceptable or competitive within their specific research field. Comparing this performance to similar studies would provide important context for evaluating the model’s effectiveness and practical applicability.

Author Response: We have revised the Discussion section accordingly (pages 21-22). Note that, after models were revisited following suggestions from reviewers, model performance improved slightly after applying SMOTE, 10-fold cross-validation, and hyperparameter tuning.

Reviewer #2: The manuscript presents an important application of classical and deep learning models for predicting E. coli contamination in household drinking water in Bangladesh using a nationally representative dataset.

Two Minor, Yet Scientifically Important Improvements

1. Clarify the rationale behind SMOTE for an already balanced dataset segment

- The reported prevalence of contamination is ~39% vs 61%. This is moderately imbalanced, not severely.

Author Response: Thank you. The outcome was indeed moderately imbalanced. Our rationale for using SMOTE was to test its ability to increase accuracy, especially for the contaminated subset. We added the rationale for SMOTE in page 10. Note that we now compare this approach with the original imbalanced dataset (with SMOTE: pages 10, and 16-19, without SMOTE: see the supplemental pages 3-4).

- Please briefly justify the use of SMOTE and discuss whether oversampling may have artificially inflated model performance or introduced bias, especially since several models showed modest AUC values (<0.70).

Author Response: As explained above, we now compare the SMOTE approach with the original imbalanced dataset (with SMOTE: pages 10, and 16-19, without SMOTE: see the supplemental pages 3-4). Additionally, please note that SMOTE was used only in the training dataset -the validation folds and the final held-out test set remained non-synthetic.

2. Add a short explanation of why AdaBoost outperformed other models

- AdaBoost achieved the highest accuracy and F1 score, but the manuscript does not interpret why it may be more suitable for this problem (e.g., handling weak learners, sensitivity to decision boundaries, feature interactions).

Author Response: This has now been added to the Discussion section (page 21).

- A 2–3 sentence interpretation would strengthen the Discussion and provide insight for practitioners choosing ML techniques for similar epidemiological datasets.

Author Response: This has now been added to the Discussion section (page 21).

Reviewer #3: This research represents an important scientific contribution in the field of public health and drinking water quality .This is achieved by applying advanced machine learning algorithms to nationally representative population data, a successful experiment in Bangladesh for predicting the risk of drinking water contamination ,but i have Nom. of comments

1-Some paragraphs are repetitive and need academic rewriting.

Author Response: Thank you for your comments. We have the entire manuscript to the best of our ability.

2-Add a comparison table between your results and the results of previous research.

Author Response: Thank you. That has now been added to supplemental files (Table 2).

3-Weaknesses in the wording of research contributions. What's new?

Author Response: We have added this information to the last paragraph of the Introduction section (page 5).

4- The statistical performance of the models was weak, and I suggest to improve by adding environmental variables related to potential pollution, such as:

• Distance from sewage treatment plants

• Population density

Author Response: We agree that the performance was weak and that is discussed in the last paragraph of the Discussion section (page 23). Additionally, please note that we now have optimized our hyperparameter turning approach based on reviewers feedback, which has increased our overall accuracy from 70.2% to 81.6% (pages 16-19).

5- The accuracy of the results is important, and this observation is crucial. These values are considered very weak for sensitive health applications. Had the researcher used appropriate deep learning algorithms for their data, the results would have been better, as these are important findings.

Author Response: Author Response: Thank you for this observation. We agree that the reported performance is insufficient for sensitive, individual-level health decision-making, and we have revised the manuscript to clarify that these models are not intended for clinical use. We included a commonly used deep learning multilayer perceptron (DL-MLP), but its performance was not substantially better than the classical ML models (page 16-19. Additionally, we now have optimized our hyperparameter turning approach based on reviewers feedback, which has increased our overall accuracy from 70.2% to 81.6% (pages 16-19).

Reviewer #4: The research was good in terms of the sources and samples used, but the practical aspect was not clear enough. The practical side needs further explanation; there should be a brief explanation of the functions used to classify the samples, as well as an explanation of how the system used in the sample analysis was evaluated.

Author Response: Thank you for your comments. We added a paragraph discussing the practical aspects of the study. Additionally, we have revised the Methods section accordingly to include the functions used (pages 8-10).

Attachment Submitted filename: Rebuttal Letter.docx

# Peer review: Decision Letter 1

**DOI:** 10.1371/journal.pone.0343606.r003

**Authors:** Sohail Saif

16 Apr 2026

PONE-D-25-52920R1

Use of machine learning to detect *Escherichia coli*  in drinking water in Bangladesh

PLOS One

Dear Dr. Nobrega,

Thank you for submitting your manuscript to PLOS ONE. After careful consideration, we feel that it has merit but does not fully meet PLOS ONE’s publication criteria as it currently stands. Therefore, we invite you to submit a revised version of the manuscript that addresses the points raised during the review process.

==============================

As discussed via email, we have rescinded the acceptance decision and returned the manuscript to you, so that you can make any necessary changes to the text and figures. Specifically, please add your updates to figure 3, figure 7, and the text of the manuscript. Once you resubmit your revised manuscript, it will be reviewed by the Academic Editor.

==============================

Please submit your revised manuscript by May 31 2026 11:59PM. If you will need more time than this to complete your revisions, please reply to this message or contact the journal office at plosone@plos.org. When you're ready to submit your revision, log on to https://www.editorialmanager.com/pone/ and select the 'Submissions Needing Revision' folder to locate your manuscript file.

Please include the following items when submitting your revised manuscript:

- A letter that responds to each point raised by the academic editor and reviewer(s). You should upload this letter as a separate file labeled 'Response to Reviewers'.
- A marked-up copy of your manuscript that highlights changes made to the original version. You should upload this as a separate file labeled 'Revised Manuscript with Track Changes'.
- An unmarked version of your revised paper without tracked changes. You should upload this as a separate file labeled 'Manuscript'.

If you would like to make changes to your financial disclosure, please include your updated statement in your cover letter. Guidelines for resubmitting your figure files are available below the reviewer comments at the end of this letter.

If applicable, we recommend that you deposit your laboratory protocols in protocols.io to enhance the reproducibility of your results. Protocols.io assigns your protocol its own identifier (DOI) so that it can be cited independently in the future. For instructions see: https://journals.plos.org/plosone/s/submission-guidelines#loc-laboratory-protocols. Additionally, PLOS ONE offers an option for publishing peer-reviewed Lab Protocol articles, which describe protocols hosted on protocols.io. Read more information on sharing protocols at https://plos.org/protocols?utm_medium=editorial-email&utm_source=authorletters&utm_campaign=protocols.

As the corresponding author, your ORCID iD is verified in the submission system and will appear in the published article. PLOS supports the use of ORCID, and we encourage all coauthors to register for an ORCID iD and use it as well. Please encourage your coauthors to verify their ORCID iD within the submission system before final acceptance, as unverified ORCID iDs will not appear in the published article. *Only*  the individual author can complete the verification step; PLOS staff *cannot*  verify ORCID iDs on behalf of authors.

We look forward to receiving your revised manuscript.

Kind regards,

Katherine Kokkinias, Ph.D.

Staff Editor, PLOS One

on behalf of

Sohail Saif, Ph.D

Academic Editor

PLOS One

Journal Requirements:

If the reviewer comments include a recommendation to cite specific previously published works, please review and evaluate these publications to determine whether they are relevant and should be cited. There is no requirement to cite these works unless the editor has indicated otherwise.

[Note: HTML markup is below. Please do not edit.]

Reviewer's Responses to Questions

**Comments to the Author**

1. If the authors have adequately addressed your comments raised in a previous round of review and you feel that this manuscript is now acceptable for publication, you may indicate that here to bypass the “Comments to the Author” section, enter your conflict of interest statement in the “Confidential to Editor” section, and submit your "Accept" recommendation.

Reviewer #1: All comments have been addressed

Reviewer #2: All comments have been addressed

Reviewer #3: All comments have been addressed

**********

2. Is the manuscript technically sound, and do the data support the conclusions?

The manuscript must describe a technically sound piece of scientific research with data that supports the conclusions. Experiments must have been conducted rigorously, with appropriate controls, replication, and sample sizes. The conclusions must be drawn appropriately based on the data presented.

Reviewer #1: Yes

Reviewer #2: Yes

Reviewer #3: Yes

**********

3. Has the statistical analysis been performed appropriately and rigorously?

Reviewer #1: Yes

Reviewer #2: Yes

Reviewer #3: Yes

**********

4. Have the authors made all data underlying the findings in their manuscript fully available?

The PLOS Data policy requires authors to make all data underlying the findings described in their manuscript fully available without restriction, with rare exception (please refer to the Data Availability Statement in the manuscript PDF file). The data should be provided as part of the manuscript or its supporting information, or deposited to a public repository. For example, in addition to summary statistics, the data points behind means, medians and variance measures should be available. If there are restrictions on publicly sharing data—e.g. participant privacy or use of data from a third party—those must be specified.

Reviewer #1: Yes

Reviewer #2: Yes

Reviewer #3: Yes

**********

5. Is the manuscript presented in an intelligible fashion and written in standard English?

PLOS ONE does not copyedit accepted manuscripts, so the language in submitted articles must be clear, correct, and unambiguous. Any typographical or grammatical errors should be corrected at revision, so please note any specific errors here.

Reviewer #1: Yes

Reviewer #2: Yes

Reviewer #3: Yes

**********

6. Review Comments to the Author

Please use the space provided to explain your answers to the questions above. You may also include additional comments for the author, including concerns about dual publication, research ethics, or publication ethics. (Please upload your review as an attachment if it exceeds 20,000 characters)

Reviewer #1: Thanks for addressing my previous comments which I think improved the paper so much. I don’t have any further comments.

Reviewer #2: The authors have adequately addressed the major and minor concerns raised during the previous round of review. Key methodological clarifications, including the justification and controlled use of SMOTE, expanded hyperparameter tuning details, improved SHAP-based feature selection explanation, and strengthened discussion on model performance, have been satisfactorily incorporated. The manuscript is now substantially improved in clarity, rigor, and transparency.

Reviewer #3: (No Response)

**********

7. PLOS authors have the option to publish the peer review history of their article (what does this mean?). If published, this will include your full peer review and any attached files.

If you choose “no”, your identity will remain anonymous but your review may still be made public.

**Do you want your identity to be public for this peer review?**  For information about this choice, including consent withdrawal, please see our Privacy Policy.

Reviewer #1: No

Reviewer #2: No

Reviewer #3: No

**********

[NOTE: If reviewer comments were submitted as an attachment file, they will be attached to this email and accessible via the submission site. Please log into your account, locate the manuscript record, and check for the action link "View Attachments". If this link does not appear, there are no attachment files.]

To ensure your figures meet our technical requirements, please review our figure guidelines: https://journals.plos.org/plosone/s/figures

You may also use PLOS’s free figure tool, NAAS, to help you prepare publication quality figures: https://journals.plos.org/plosone/s/figures#loc-tools-for-figure-preparation.

NAAS will assess whether your figures meet our technical requirements by comparing each figure against our figure specifications.

# Author comment: Author response to Decision Letter 2

**DOI:** 10.1371/journal.pone.0343606.r004

28 Apr 2026

N/A

Attachment Submitted filename: response.pdf

# Editor report: Decision Letter 2

**DOI:** 10.1371/journal.pone.0343606.r005

**Authors:** Sohail Saif

10 May 2026

Use of machine learning to detect *Escherichia coli*  in drinking water in Bangladesh

PONE-D-25-52920R2

Dear Dr. Nobrega,

We’re pleased to inform you that your manuscript has been judged scientifically suitable for publication and will be formally accepted for publication once it meets all outstanding technical requirements.

Within one week, you’ll receive an e-mail detailing the required amendments. When these have been addressed, you’ll receive a formal acceptance letter and your manuscript will be scheduled for publication.

An invoice will be generated when your article is formally accepted. Please note, if your institution has a publishing partnership with PLOS and your article meets the relevant criteria, all or part of your publication costs will be covered. Please make sure your user information is up-to-date by logging into Editorial Manager at Editorial Manager® and clicking the ‘Update My Information' link at the top of the page. For questions related to billing, please contact billing support.

If your institution or institutions have a press office, please notify them about your upcoming paper to help maximize its impact. If they’ll be preparing press materials, please inform our press team as soon as possible -- no later than 48 hours after receiving the formal acceptance. Your manuscript will remain under strict press embargo until 2 pm Eastern Time on the date of publication. For more information, please contact onepress@plos.org.

Kind regards,

Sohail Saif, Ph.D

Academic Editor

PLOS One

Additional Editor Comments (optional):

Response is satisfactory.

Reviewers' comments:

# Editor report: Acceptance letter

**DOI:** 10.1371/journal.pone.0343606.r006

**Authors:** Sohail Saif

PONE-D-25-52920R1

PLOS One

Dear Dr. Nobrega,

I'm pleased to inform you that your manuscript has been deemed suitable for publication in PLOS One. Congratulations! Your manuscript is now being handed over to our production team.

At this stage, our production department will prepare your paper for publication. This includes ensuring the following:

* All references, tables, and figures are properly cited

* All relevant supporting information is included in the manuscript submission,

* There are no issues that prevent the paper from being properly typeset

You will receive further instructions from the production team, including instructions on how to review your proof when it is ready. Please keep in mind that we are working through a large volume of accepted articles, so please give us a few days to review your paper and let you know the next and final steps.

Lastly, if your institution or institutions have a press office, please let them know about your upcoming paper now to help maximize its impact. If they'll be preparing press materials, please inform our press team within the next 48 hours. Your manuscript will remain under strict press embargo until 2 pm Eastern Time on the date of publication. For more information, please contact onepress@plos.org.

You will receive an invoice from PLOS for your publication fee after your manuscript has reached the completed accept phase. If you receive an email requesting payment before acceptance or for any other service, this may be a phishing scheme. Learn how to identify phishing emails and protect your accounts at https://explore.plos.org/phishing.

If we can help with anything else, please email us at customercare@plos.org.

Thank you for submitting your work to PLOS ONE and supporting open access.

Kind regards,

PLOS ONE Editorial Office Staff

on behalf of

Dr. Sohail Saif

Academic Editor

PLOS One
